# Integrating Humidity‐Resistant and Colorimetric COF‐on‐MOF Sensors with Artificial Intelligence Assisted Data Analysis for Visualization of Volatile Organic Compounds Sensing

**DOI:** 10.1002/advs.202411621

**Published:** 2025-01-31

**Authors:** Qin Ouyang, Yanna Rong, Gaofan Xia, Quansheng Chen, Yujie Ma, Zhonghua Liu

**Affiliations:** ^1^ School of Food and Biological Engineering Jiangsu University Zhenjiang 212013 P. R. China; ^2^ Tea Industry Research Institute Fujian Eight Horses Tea Co. Ltd Quanzhou 362442 P. R. China; ^3^ Department of Chemistry University of Manchester Manchester M13 9PL UK; ^4^ National Research Center of Engineering and Technology for Utilization of Botanical Functional Ingredients Hunan Agricultural University Changsha 410128 P. R. China

**Keywords:** AI‐assisted data analysis, artificial olfactory arrays, COF‐on‐MOF materials, colorimetric sensors, visualization of VOCs sensing

## Abstract

Direct visualization and monitoring of volatile organic compounds (VOCs) sensing processes via portable colorimetric sensors are highly desired but challenging targets. The key challenge resides in the development of efficient sensing systems with high sensitivity, selectivity, humidity resistance, and profuse color change. Herein, a strategy is reported for the direct visualization of VOCs sensing by mimicking human olfactory function and integrating colorimetric COF‐on‐MOF sensors with artificial intelligence (AI)‐assisted data analysis techniques. The Dye@Zeolitic Imidazolate Framework@Covalent Organic Framework (Dye@ZIF‐8@COF) sensor takes advantage of the highly porous structure of MOF core and hydrophobic nature of the COF shell, enabling highly sensitive colorimetric sensing of trace number of VOCs. The Dye@ZIF‐8@COF sensor exhibits exceptional sensitivity to VOCs at sub‐parts per million levels and demonstrates excellent humidity resistance (under 20–90% relative humidity), showing great promise for practical applications. Importantly, AI‐assisted information fusion and perceptual analysis greatly promote the accuracy of the VOCs sensing processes, enabling direct visualization and classification of seven stages of matcha drying processes with a superior accuracy of 95.74%. This work paves the way for the direct visualization of sensing processes of VOCs via the integration of advanced humidity‐resistant sensing materials and AI‐assisted data analyzing techniques.

## Graphical abstract

The direct visualization of sensing processes of volatile organic compounds via the integration of AI‐assisted data analyzing techniques and colorimetric COF‐on‐MOF sensors is reported. This work shows excellent sensing sensitivity and selectivity, representing a universal platform for fabricating sensing systems for the direct visualization and identification of various gas‐sensing processes.

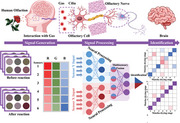

## 1 Introduction

The ability to effectively observe different objects and to accurately recognize targets is a perceptive skill that humans have developed during their evolutionary history.^[^ ^1^ ^]^ Humans have five primary senses: sight, hearing, smell, taste, and touch, which organically and individually function to ensure that they interact and react to their surroundings.^[^ ^2^ ^]^ Among these senses, olfaction is highly specialized for detecting volatile organic compounds (VOCs).^[^ ^3^ ^]^ VOCs activate a combined response pattern of different olfactory receptors, triggering specific nerve impulses that culminate in the perception and recognition of VOCs.^[^ ^4^ ^]^ Inspired by human olfaction, artificial olfaction conceives multigas sensor arrays for VOCs fingerprint pattern recognition.^[^ ^5^, ^6^ ^]^ Traditional commercial metal oxide sensors are widely employed as general‐purpose sensors, but these sensors can be influenced intensively by humidity interaction, causing signal drifts and limiting their ability to meet detection requirements for specific target analytes.^[^ ^7^ ^]^ Compared to traditional metal oxide sensors, the colorimetric sensor arrays (CSAs) exhibit specific response performance to analytes, thereby offering enhanced sensitivity and selectivity. CSAs are suitable for a variety of fields, including biomedical diagnosis,^[^ ^8^ ^]^ smart farms,^[^ ^9^ ^]^ environment monitoring,^[^ ^10^ ^]^ food storage monitoring,^[^ ^11^ ^]^ and hazard detection.^[^ ^12^ ^]^ Despite extensive research on CSAs, their limitations such as humidity dependence and lack of sensitivity prevent it from being highly specialized to mimic the human olfactory system.^[^ ^13^ ^]^

The uniform loading of dyes within porous material and subsequent hydrophobic modification on the pore surface can effectively solve the humidity interference and poor sensitivity in CSA. Metalloporphyrins are almost ideal dye choices for the detection of VOCs because they can provide coordination sites for target analytes.^[^ ^3^, ^6^ ^]^ They are also highly conjugated p‐type organic semiconductors that can induce charge transfer between their isolated π‐systems and various oxidizing or reducing VOCs. The interactions between the dyes and VOC analytes can cause profuse color change, which is desired for the development of colorimetric sensors for VOCs. Metal‐organic frameworks (MOFs) composed of metal ions and organic linkers are a new class of porous materials.^[^ ^14^, ^15^ ^]^ MOFs offer unique advantages in sensing applications because of their highly porous and tunable structures, offering sufficient space for rapid diffusion of analytes and numerous active sites for interaction.^[^ ^16^, ^17^, ^18^, ^19^ ^]^ This not only enables the pre‐concentration of analytes within the MOF pores, but also prevents the dyes from aggregation, thus enhancing sensing sensitivity and selectivity. Among all the MOF materials, zeolite imidazolate framework‐8 (ZIF‐8) is widely reported for sensing applications due to its unique porous structure and large surface area, which can enhance the sensing performance.^[^ ^20^, ^21^ ^]^ Additionally, its chemical and thermal stability ensures good performance under various conditions, enabling the development of versatile sensors for specific targets. However, Dye@ZIF‐8 composites often suffer from their poor sensing performance in high‐humidity environments. Surface hydrophobic modification of Dye@ZIF‐8 materials is considered a useful approach to improve their water‐resistance performance. Covalent organic frameworks (COFs), with the advantageous nature of their hydrophobicity and porous microenvironment, serve as ideal hydrophobic modification materials.^[^ ^22^, ^23^, ^24^ ^]^ As a result, modifying Dye@ZIF‐8 sensors with hydrophobic COF layers can be a promising strategy to prepare Dye@ZIF‐8@COF sensors for highly sensitive and selective detection of VOCs released in various chemical processes.

Recent advancements in artificial intelligence (AI) have significantly enhanced image‐based pattern recognition capabilities.^[^ ^25^, ^26^ ^]^ One promising development is the AI‐assisted Biological Olfactory Learning Architecture (BOLA) which emulates the hierarchical fusion of multiple olfactory signals observed in the brain. This architecture comprises neural networks designed to replicate the intricate processing hierarchy of olfactory signals. Specifically, 1D convolutional neural networks (1D‐CNN) are employed for early‐stage olfactory information processing. AI‐assisted 1D‐CNN are chosen due to their ability to mimic the local receptive field function observed in biological nervous systems, thus effectively simulating the initial processing of olfactory information in the primary areas of brains.^[^ ^27^ ^]^ This approach holds great promise for advancing our understanding of olfactory perception and broadening its applications in various fields such as environmental monitoring, agriculture, and healthcare.^[^ ^28^, ^29^, ^30^ ^]^ As a result, integrating colorimetric COF‐on‐MOF sensors with AI‐assisted data analysis techniques can be a promising strategy for the identification and direct visualization of VOCs sensing processes.

In this work, we have innovatively developed a CSA system based on colorimetric Dye@ZIF‐8@COF composites to accurately monitor the trace amount of VOCs released in matcha drying processes. The COF‐on‐MOF sensor shows superior sensitivity to various VOCs at sub‐parts per million levels (< 1 ppm) and demonstrates excellent resistance to humidity (20‐90% relative humidity). In the meantime, we employ AI‐assisted 1D‐CNN as the framework for information processing to enhance the accuracy of sensing processes. By combining the environmentally friendly, humidity‐independent, and highly sensitive Dye@ZIF‐8@COF sensor with AI‐assisted data analysis techniques, we realize the direct visualization and monitoring of the VOCs sensing in seven stages of matcha drying processes with excellent accuracy of 95.74%. This study provides a potentially promising solution to the monitoring and visualization of various gas sensing processes via integrating versatile colorimetric sensors with AI‐assisted data analyzing techniques.

## 2 Results and Discussion

### 2.1 Preparation and Characterization of the Colorimetric COF‐on‐MOF Sensors

**Figure 1:**
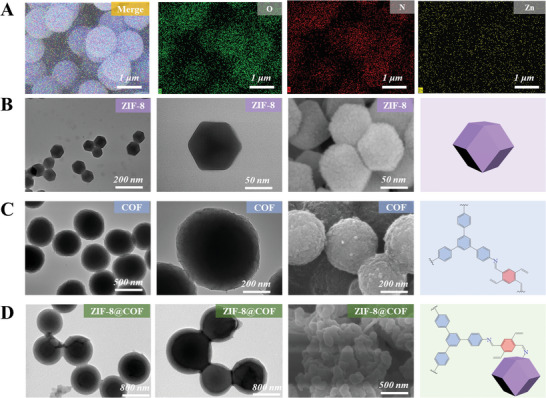
Morphological characterization of the COF‐on‐MOF sensors. A) Scanning electron microscopy (SEM) and EDS mapping images of ZIF‐8@COF composite. SEM and transmission electron microscope (TEM) images of B) NH_2_‐ZIF‐8, C) COF, and D) ZIF‐8@COF materials.

**Figure 2:**
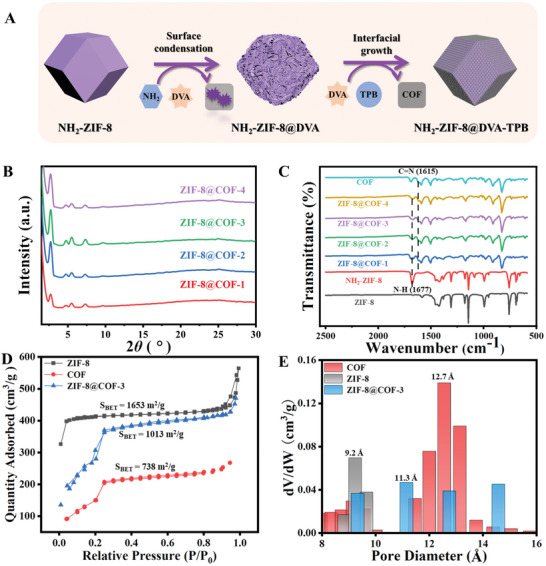
A) Schematic illustration for the preparation of the core‐shell ZIF‐8@COF composites. B) XRD patterns of ZIF‐8@COF materials. C) FT‐IR spectra of the composite materials. D) N_2_ adsorption‐desorption isotherms, and E) pore‐size distribution of the composite materials.

The COF‐on‐MOF sensor was prepared by coating a hydrophobic COF shell on the pristine MOF layer. A Zn‐based MOF (‐NH_2_ functionalized ZIF‐8, abbreviated as NH_2_‐ZIF‐8 or ZIF‐8) was chosen as the core due to its high stability and ‐NH_2_ group, which can promote the growth of the COF shell on the MOF material. We selected a representative COF owing to its hydrophobic properties and the availability of its simple synthetic method at room temperature. **Figures** **1** and **2**A depict the synthetic process of the hybrid material (ZIF‐8@COF), with the formation of a COF shell layer achieved by utilizing a condensation reaction of 2,5‐divinylterephthalaldehyde (DVA) and 1,3,5‐tris (4‐aminophenyl) benzene (TPB). Basically, DVA was coupled to NH_2_‐ZIF‐8 with the formation of an imide group (─C═N), followed by the covalent attachment of TPB to the unreacted aldehyde group (─CHO) of DVA.

To investigate the structure of the composite materials, various characterization techniques were employed, including scanning electron microscopy (SEM), transmission electron microscope (TEM), X‐ray diffraction (XRD), N_2_ adsorption‐desorption isotherms, X‐ray photoelectron spectroscopy (XPS) and Fourier transform infrared (FT‐IR) spectroscopy. Original NH_2_‐ZIF‐8 exhibited a polyhedron shape and smooth surface (particle size ≈200 nm) (Figure 1B), whereas COF had a homogeneous spherical morphology with a size of ≈450 nm (Figure 1C). After coating, the MOF core was decorated by a COF shell (Figure 1D). Energy dispersive spectroscopy (EDS) mapping images of ZIF‐8@COF‐3 show a uniform distribution of the elements Zn, N, and O (Figure 1A), indicating the successful preparation of the composite material.

In the XRD patterns (Figure 2B; Figure S1A, Supporting Information), both NH_2_‐ZIF‐8 and COF exhibit commendable crystallinity with their distinct diffraction peaks aligning with previously reported data. The XRD patterns of ZIF‐8@COF retain the unique diffraction peaks of both materials, indicating the successful preparation of the material. FT‐IR spectra of the ZIF‐8@COF material show the characteristic peaks at 1615 and 1677 cm^−1^ (Figure 2C), originating from the C═N stretching band and N─H stretching band of the COF and MOF materials, respectively. As shown in Figure S1B (Supporting Information), the zeta potential of NH_2_‐ZIF‐8 alone is positive, while that of COF is negative. Upon coating the COF shell, the zeta potential of NH_2_‐ZIF‐8 changed from positive to negative, indicating the successful coating of the COF layer on the MOF surface. The porous structure of the materials was investigated via N_2_ adsorption‐desorption isotherms (Figure 2D). ZIF‐8@COF‐3 displayed a Brunauer‐Emmett‐Teller (BET) surface area of 1013 m^2^ g^−1^, which is between the BET surface areas of NH_2_‐ZIF‐8 (1653 m^2^ g^−1^) and COF material (738 m^2^ g^−1^). This insight reveals that the COF has formed on the external surface of the MOF material without causing pore obstruction of the ZIF‐8 material. The ZIF‐8@COF‐3 composite effectively retains the pore size distribution of both NH_2_‐ZIF‐8 (9.2 Å) and COF (12.7 Å) (Figure 2E). The retention of the porous structure can contribute to the pre‐concentration of analytes and enhance the sensing sensitivity. The N 1s XPS spectrum for ZIF‐8@COF‐3 is analyzable into two distinct peaks (Figure S1C, Supporting Information), corresponding to the C─N (398.7 eV) bond in NH_2_‐ZIF‐8 and the C═N (398.3 eV) bond in the COF material,^[^ ^31^, ^32^ ^]^ underscoring the transformation of amine to imine functionalities upon the COF formation. Overall, the complementary characterization techniques collectively confirm the successful preparation of composite materials.^[^ ^33^, ^34^ ^]^

### 2.2 Design and Preparation of Colorimetric Sensor Arrays Based on Humidity Resistant Dye@ZIF‐8@COF Composites

**Figure 3:**
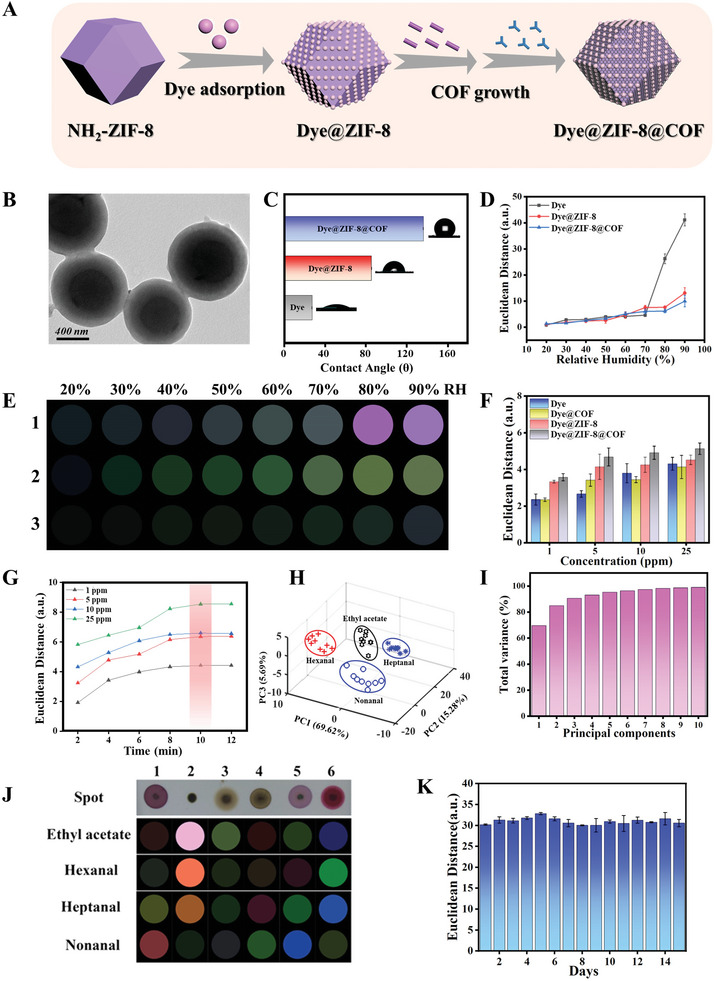
Fabrication of the Dye@ZIF‐8@COF material for VOCs sensing. A) Schematic diagram of the preparation of Dye@ZIF‐8@COF composite material. B) TEM image of the Dye@ZIF‐8@COF material. C) Water contact angle images of the colorimetric sensors. D) Euclidean distance values and E) Colorful difference images of Dye (1), Dye@ZIF‐8 (2), and Dye@ZIF‐8@COF (3) in response to different relative humidity levels. F) ED values and color differential profiles for the colorimetric sensors to ethyl acetate with different concentrations. G) Response kinetics of the Dye@ZIF‐8@COF material to ethyl acetate with different concentrations over time. H) PCA plot of four VOCs and control based on the response of five parallel trials, using the first three principal components. I) Corresponding PCA results. J) Color difference images of the colorimetric Dye@ZIF‐8@COF sensors for identification and direct visualization of various VOCs sensing at 10 ppm (Spot 1–6 represent the Dye@ZIF‐8@COF sensors incorporating 6 different dyes listed in Table S1, Supporting Information). K) Stability test of Dye@ZIF‐8@COF sensor for VOCs sensing.

**Figure 4:**
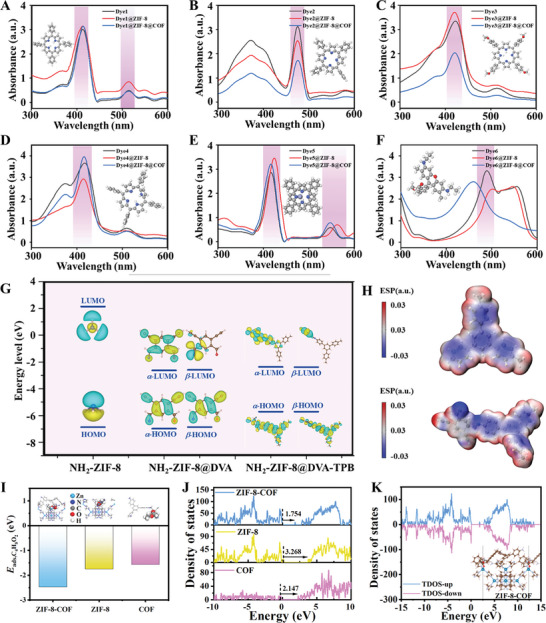
A–F) UV–vis absorption spectra of six kinds of dyes, Dye@ZIF‐8 and Dye@ZIF‐8@COF materials. Insets: ball‐and‐stick model of A) 5, 10, 15, 20‐Tetraphenyl‐21H, 23H‐porphin, B) 5, 10, 15, 20‐Tetraphenyl‐21H, 23H‐porphine manganese (III) chloride, C) 5, 10, 15, 20‐Tetrakis (4‐methoxyphenyl) ‐21H, 23H‐porphine iron (III) chloride, D) 5, 10, 15, 20‐Tetraphenyl‐21H, 23H‐porphine iron (III) chloride, E) 5, 10, 15, 20‐Tetraphenyl‐21H, 23H‐porphine zinc and F) basic red. G) Simulation results for the HOMO and LUMO energy levels. H) Electrostatic potentials of the sensing materials. I) Adsorption energy results of ZIF‐8, COF, and ZIF‐8@COF for ethyl acetate. J) and K) Density of states of ZIF‐8, COF, and ZIF‐8@COF.

The fabrication of Dye@ZIF‐8@COF composites involves a two‐step approach (**Figure** **3**A). Initially, NH_2_‐ZIF‐8 with high surface area was selected to effectively adsorb the dye molecules by direct solution adsorption technique. Subsequently, the Dye@ZIF‐8 powder was coated with a COF shell (Figure 3B) to achieve hydrophobic properties. Combining the highly porous structure of MOF core and humidity resistant nature of the COF shell, Dye@ZIF‐8@COF composites are superior gas‐sensitive materials for VOCs sensing. Typically, desolvated NH_2_‐ZIF‐8 powder was placed in a dye solution to gradually adsorb dye molecules. After the adsorption of dye molecules, the ZIF‐8 powder showed an obvious color change. The adsorption phenomenon was further confirmed by UV–vis absorption spectroscopy (**Figure** **4**). The COF shell was then coated on the Dye@ZIF‐8 material (see Methods Section for the details). The prepared Dye@ZIF‐8@COF composites exhibited enhanced hydrophobicity with a contact angle of 130° in contrast to the hydrophilic dyes with a contact angle of 30° (Figure 3C). To evaluate COF shell's contribution to humidity resistance, experiments were conducted with three variants including dye alone, Dye@ZIF‐8, and Dye@ZIF‐8@COF. These samples were drop‐casted on substrates and subjected to humidity tests in a controlled chamber. Following a 12‐min exposure to varying relative humidity (RH) levels ranging from 20% to 90%, the Dye@ZIF‐8@COF composites exhibited minimal Euclidean Distance (ED) values across all RH levels (Figure 3D; Figure S3, Supporting Information), underscoring their superior humidity resistant property due to the hydrophobic COF layer. The colorimetric change (ΔR, ΔG, ΔB) in response to water vapor for each material was quantified by comparing images before and after humidity exposure, and the results were visually represented through color differential mapping (Figure 3E; Figure S4, Supporting Information). Notably, the hydrophobic Dye@ZIF‐8@COF composites showed negligible response to water vapor, even under extremely high humidity conditions (90% RH). In contrast, both the dye and Dye@ZIF‐8 samples displayed progressively stronger colorimetric changes as the humidity exceeded 30% RH. The humidity resistance of these materials was further quantified using the ED in the RGB color space, where a higher ED indicates a more pronounced reaction to moisture. ED is the distance of the straight line between two points in the RGB color (red, green, blue) space ED = $\sqrt{▵R^{2}+▵G^{2}+▵B^{2}}.$

The interaction of three colorimetric sensors (dye, Dye@ZIF‐8, and Dye@ZIF‐8@COF) with varying concentrations of ethyl acetate is depicted in Figure 3F and Figure S5 (Supporting Information). Analysis of both ED values and color difference images for Heptanal, Hexanal, and Nonanal demonstrates that Dye@ZIF‐8 and Dye@ZIF‐8@COF materials possess superior VOC detection capabilities compared to the dye‐based sensor alone (Figures S6–S8, Supporting Information). The detection time was fine‐tuned by subjecting colorimetric sensor arrays to ethyl acetate with concentrations of 1, 5, 10, and 25 ppm, revealing that the average ED values for six sensor spots essentially reach a plateau at 10 min (Figure 3G). Moreover, the sensor's discernibility to four distinct VOCs exhibited a clear concentration correlation (Figures S9 and S10, Supporting Information). As shown in Figure 3J, the colorimetric Dye@ZIF‐8@COF sensors show unique and identifiable color changes and response patterns upon interactions with each type of VOCs (at a concentration of 10 ppm), affirming the sensor array's capability to distinguish between multiple VOCs. This also enables the identification and direct visualization of various VOCs sensing processes, which is highly desired for the development of colorimetric VOC sensors. Principal Component Analysis (PCA) was applied to the accumulated data at various concentrations. PCA results from the dataset of four VOCs at a concentration of 10 ppm can clearly separate the VOCs as the first three principal components (PCs) account for >90% of the total variance. This indicates a robust classification performance (Figure 3H,I). The Dye@ZIF‐8@COF sensors show low limit of detection (LOD) values of below 1 ppm for VOCs sensing (Table S2, Supporting Information), indicating their excellent sensing performance. In addition, the stability of the Dye@ZIF‐8@COF sensor was investigated (Figure 3K; Figures S12–S15, Supporting Information). As shown in Figures S12–S15 (Supporting Information), the Dye@ZIF‐8@COF sensors exhibit good stability at room temperature for 15 days.

### 2.3 Colorimetric Dye@ZIF‐8@COF Sensor Arrays for the Recognition and Direct Visualization of VOCs Sensing Processes

Since Dye@ZIF‐8@COF composites exhibit strong moisture resistance and sensitivity in VOCs sensing, we devised an artificial olfactory array incorporating six distinct types of Dye@ZIF‐8@COF composites for the purpose of detecting and recognizing matcha VOCs (Figure S2, Supporting Information). The discriminatory response of this sensor array is characterized by its high selectivity, which relies on cross‐reactive interactions between chemically reactive dyes and target analytes. Six different types of dyes listed in Table S1 (Supporting Information) were used for the preparation of six colorimetric Dye@ZIF‐8@COF sensors. In addition, Dye@ZIF‐8 and Dye@ZIF‐8@COF composites show similar UV–vis absorption spectra (Figure 4A–F) to the pristine dyes, indicating that the optical properties of the dyes are well retained in the pores of the composite materials.

The electrostatic potential (ESP), lowest unoccupied molecular orbital (LUMO), and highest occupied molecular orbital (HOMO) of NH_2_‐ZIF‐8 and COF materials were calculated using the Gaussian distribution method (Figure 4G; Figure S16, Supporting Information). The ESP values of the composites are shown in Figure 4H. The red area represents the electrophilic region, indicating the tendency to accept electrons; and the blue (negative) area represents the nucleophilic region, indicating the tendency to give electrons. After the COF shell coating, the ZIF‐8@COF material exhibits a reduced HOMO‐LUMO energy gap compared to pristine NH_2_‐ZIF‐8 (Table S4, Supporting Information). This reduction may enhance the material's responsiveness to VOCs and improve its sensing sensitivity. The calculated adsorption energy of VOC (ethyl acetate) molecule on ZIF‐8@COF material is more negative than those for ZIF‐8 and COF materials (Figure 4I), indicating the stronger adsorption of VOCs onto ZIF‐8@COF material. In addition, the density of states results show a smaller band gap of ZIF‐8@COF material compared with the ZIF and COF materials (Figure 4J,K). This indicates a more conductive electronic structure of the ZIF‐8@COF material, which could enhance the electron transfer and benefit the sensing performance of the former one.^[^ ^35^, ^36^ ^]^ Overall, the density functional theory calculations are consistent with the experimental results, confirming the enhanced sensing performance of the ZIF‐8@COF material.

In the context of VOCs sensing, the porous and hydrophobic COF layer of the colorimetric sensors facilitates the access of VOCs to the dye for colorimetric reaction while concurrently preventing the penetration of water molecules from the surrounding environment. The sensor arrays' exceptional resistance to humidity was evidenced by their minimal reaction across relative humidities (20–90% RH), in contrast to sensor arrays prepared with dye alone and Dye@ZIF‐8 materials (Figure 3E; Figure S4, Supporting Information). To evaluate the multiplexed sensing capabilities of the colorimetric Dye@ZIF‐8@COF sensor arrays, experimental tests were performed to identify VOCs during the matcha drying process. The headspace solid‐phase‐microextraction coupled gas‐chromatography and mass‐spectrometry (HS‐SPME‐GC‐MS) method was applied for the comprehensive analysis of the VOCs released from the samples (from Stage‐1 to Stage‐7). The series of 220 VOCs found in samples were identified and quantified (Figure S17, Supporting Information), and the characteristic HS‐SPME‐GC‐MS data were used to distinguish the VOCs released in seven stages of the matcha drying process (Figure S18A, Supporting Information). The results of the VOCs hierarchical clustering analysis (HCA) model (Figure S18B, Supporting Information) analyzed by HS‐SPME‐GC‐MS were found to be consistent with orthogonal projections to latent structures discriminant analysis (OPLS‐DA) (R^2^Y = 0.999; Q^2^ = 0.996). Response permutation tests were used to determine the statistical significance of these high‐predictor variable models (200 permutation tests) (Figure S18C, Supporting Information). The discriminant model contained 220 VOCs, with 53 of them having greater than 1.0 VIP values (Table S5 and Figure S18D, Supporting Information), indicating their significant contribution to the model. Principal component analysis was used for dimensionality reduction of 53 VOCs.

As shown in Table S6 (Supporting Information), the first six principal components (PCs) contributed 97.52% of the total variance, whereas the eigenroots were all >1, indicating that the first six PCs represent substantial original information of 53 VOCs. The PCs extracted from the PCA were used to obtain VOCs with higher factors of loading values (Figure S18E,F, Supporting Information). The loadings of each quality index in the six PCs were used to investigate its representative index (Tables S7 and S8, Supporting Information). Then correlation analysis was performed for representative VOCs of each PC (Figure S18G, Supporting Information). Similarly, the 20 VOCs selected for the comprehensive analysis were used as representative samples of the first six PCs to perform cluster analysis (Table S9 and Figure S18H, Supporting Information). Eventually, ethyl acetate, hexanal, heptanal, and nonanal were identified as the characteristic VOCs of the samples by performing ANOVA (analysis of variance) and correlation analysis on the 20 characteristic VOCs (Figures S18I,S19,S20, and Table S10, Supporting Information). Moreover, the sensor's visible reaction to these four VOCs demonstrated a dependency on concentration levels (Figure S21, Supporting Information).

### 2.4 Accurate Recognition and Direct Visualization of VOCs Sensing in 7 Stages of Matcha Drying Processes

A total of 140 samples (20 samples × 7 stages) from the matcha drying process were used for sensing. The samples were positioned in a sealed vial with the Dye@ZIF‐8@COF sensor arrays, where the released VOCs engaged with the sensor array for a duration of 8 min to induce a colorimetric response. The sensor array's reactions to the VOCs released in 7 stages of the matcha drying process with the corresponding response patterns are delineated in Figure S22 (Supporting Information). The mean ED values reflecting the sensor array's responses (Figure S23, Supporting Information) revealed varying levels from Stage 1 to Stage 7. The PCA was performed to visualize the clustering potential between different matcha drying stages and attempt to extract valuable information from the original data. Figure S24A (Supporting Information) presented a score graph of the first three PCs with PC1, PC2, and PC3. It can be observed that despite the apparent clustering pattern in identical samples, the samples crossed each other between different categories resulting in the unsatisfactory identification effect. The first three loadings and scores, which explained 56.79%, 11.15%, and 7.23% of the spectral information, were also shown in Table S11 (Supporting Information), respectively. This suggested that the load peak has a close correlation with the volatile components in matcha. Therefore, PCA plays an important role in data compression and extraction. The first 10 PCs also contributed 96.85% of the total variance, showing that they substantially represented original data from the indicators.

The result of the linear discriminant analysis (LDA) indicated that the identification rates of the calibration and prediction sets of the VOCs released in the matcha drying process are 86.02% and 80.85% with PCs = 7 (Figure S24B, Supporting Information). The Stage‐1 and Stage‐2 samples were identified with the majority of errors (Figure S24C, Supporting Information). The support vector machine (SVM) model demonstrated an identification rate of 98.92% in the calibration set and 93.62% in the prediction set (Table S12, Supporting Information). The logarithm of *c* and *g* was adopted as the coordinate axis to accurately respond to the interaction validation between parameters. In the case of the highest identification rate with *c* = 4 and *g* = 0.18, respectively (Figure S24D, Supporting Information). In addition, superior to the results obtained with a linear model, the SVM model only showed a few classification errors for the VOCs released in the matcha drying process (Figure S24E,F, Supporting Information). The classification and regression tree (CART) model is demonstrated in Figure S24G (Supporting Information). The tree was split according to whether the variable value was greater than or less than or equal to 1.278 as the root node, and finally, 7 endpoint tree branch diagrams were generated. The prediction set of the CART model contained 3 misclassified samples with a 93.48% correct identification rate (Figure S24H,I, Supporting Information).

### 2.5 Recognition Performance of the AI‐Assisted BO Algorithm

**Figure 5:**
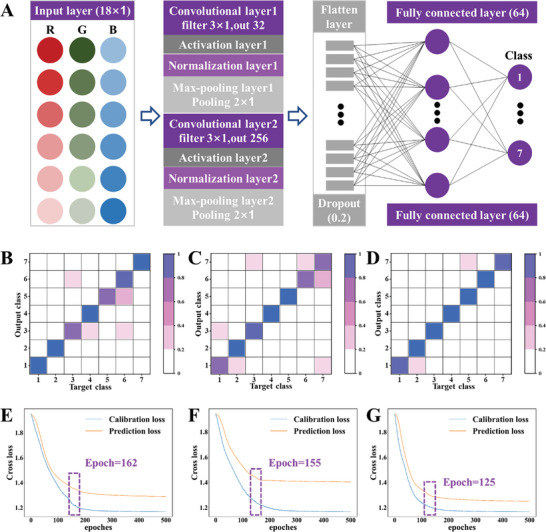
A) AI‐assisted 1D‐CNN for data analysis with superior accuracy. Scheme showing how tactile and olfactory information is processed in the AI‐assisted 1D‐CNN associated learning architecture. B–D) Confusion matrix of the optimal discriminant models for the monitor and visualization of the VOCs released in the matcha drying process by integrating AI‐assisted 1D‐CNN and Dye@ZIF‐8@COF sensors [(B) Dye, (C) Dye@ZIF‐8 and (D) Dye@ZIF‐8@COF materials]. E–G) Convergence speed plots of 1D‐CNN models based on (E) Dye, (F) Dye@ZIF‐8, and (G) Dye@ZIF‐8@COF.

A total of 140 samples (20 samples× 7 stages) from the matcha drying process were used for modeling. These samples (n = 140) were reacted with Dye, Dye@ZIF‐8, and Dye@ZIF‐8@COF sensors. The feature images of the sensors, which had been subjected to variable extraction, were used as inputs for network training following their reaction with matcha samples of different drying stages. A CrossEntropyLoss function was employed as a loss function during the training of the AI‐assisted 1D‐CNN model for the classification of matcha drying processes. The Adaptive Momentum Estimation optimizer was utilized to optimize the loss function and update the model parameters, with a learning rate of 0.01, a batch size of 30, and a training period of 200 rounds (**Figure** **5**A).

Class Activation Mapping (CAM) was used to generate a heat map for reacting to the mean weights of the convolutional layers.^[^ ^37^, ^38^ ^]^ Compared with Dye (Figure S25A, Supporting Information) and Dye@ZIF‐8 (Figure S25B, Supporting Information), Dye@ZIF‐8@COF (Figure S25C, Supporting Information) shows more important features with mean weights over 0.5, especially for the R‐values and G‐values. As a result, the AI‐assisted 1D‐CNN recognition models are able to make more accurate predictions using Dye@ZIF‐8@COF features. Figure 5 illustrates the distribution of prediction results (Figure 5B–D) and the trend of mean squared error (MSE) loss (Figure 5E–G) generated by the AI‐assisted 1D‐CNN model. The results indicate that the identification rates of the prediction set of the VOCs released in the matcha drying process are 89.36% (Table S12, Supporting Information). Most of the discrimination errors occurred in the Stage‐4, Stage‐5, and Stage‐6 samples (Figure 5B). The AI‐assisted 1D‐CNN model based on the Dye@ZIF‐8 sensor showed that the identification rates of the prediction sets reached 85.11% (Table S12, Supporting Information), which were superior to the results obtained with the LDA, SVM, and CART models (Figure 5C). As illustrated in Figure 5D, the prediction set of the AI‐assisted 1D‐CNN model based on the Dye@ZIF‐8@COF sensor shows a correct identification rate of 95.74% and the lowest MSE, confirming the accuracy of this method.

## 3 Conclusion

The development of new technologies and materials to enable the monitoring and direct visualization of VOCs sensing processes is an important target. We have integrated COF‐on‐MOF sensors with AI‐assisted data analyzing techniques to realize the direct visualization of sensing processes of VOCs released in matcha drying processes. Benefiting from the synergies between porous MOF core and hydrophobic COF shell, the Dye@ZIF‐8@COF sensors exhibit outstanding moisture resistance (negligible response to various humidity levels of 20–90% RH) and excellent sensitivity to VOCs at sub‐ppm levels (< 1 ppm). We conduct an AI‐assisted technique to analyze the data obtained from six types of Dye@ZIF‐8@COF materials for VOCs sensing and find that the AI‐assisted BO‐based architecture could identify the VOCs released in seven stages of matcha drying process with excellent accuracy of 95.74%. Our system demonstrates great promise for the direct visualization and identification of gas sensing processes by integrating AI‐assisted data analysis techniques with COF‐on‐MOF sensors.

## 4 Experimental Section

### Synthesis of ZIF‐8@COF Material

NH_2_‐ZIF‐8 (30 mg) and quantities of 2,5‐divinylterephthalaldehyde (DVA) (11 mg) were solubilized in 5 mL of acetonitrile (ACN), after which varying volumes of 12 m HAc (0.1, 0.3, 0.5, 0.7 mL) were integrated. Four different concentrations of HAc were investigated to tune the synthetic processes of ZIF‐8@COF composites, with the formation of four products denoted as ZIF‐8@COF‐1, ZIF‐8@COF‐2, ZIF‐8@COF‐3, and ZIF‐8@COF‐4, respectively. It was found that the amount of HAc can be modified to regulate the formation of the COF shell surrounding the MOF core and the optimal parameter of 0.5 mL of HAc was identified. Subsequently, the solution was subjected to ultrasonication for 5 min. Thereafter, 1,3,5‐tris(4‐aminophenyl) benzene (TPB) (14 mg), pre‐dissolved in 1 mL of ACN, was incrementally added over a period of 30 min. The concoction was gently stirred for 1 h and then left to stand at ambient temperature for 72 h. The resultant yellow precipitate was isolated through centrifugation at 9000 rpm for 15 min and underwent successive washing three times with tetrahydrofuran (THF) and ethanol. The collected samples were then dried in a vacuum for 24 h at room temperature.

### Synthesis of Dye@ZIF‐8@COF Sensors

A solution was prepared by dissolving dye (10 mg) in 40 mL of ethanol and 30 mg of NH_2_‐ZIF‐8 was subsequently added. The Dye@ZIF‐8 mixture was isolated using centrifugation at 9000 rpm for 20 min, followed by triple washing with ethanol, and then vacuum‐dried for 12 h at ambient temperature. Afterward, the mixture underwent ultrasonication for 10 min, and then 11 mg of DVA and 0.5 mL of HAc (12 m) were added. After sonication for 10 min, 14 mg of TPB dissolved in 1 mL of acetonitrile was gradually added over 30 min. This blend was gently stirred for 1 h and then left undisturbed at room temperature for a duration of 72 h. The Dye@ZIF‐8@COF product was obtained through centrifugation at 9000 rpm for 20 min, followed by sequential washing three times with THF and ethanol. The sample was finally dried under vacuum for 24 h at room temperature.

### Preparation of Dye@ZIF‐8@COF Sensor Arrays

The Dye@ZIF‐8@COF (50 mg) was suspended in 1 mL of ethanol or dichloromethane using ultrasound to prepare a CSA. The suspension solution was then dropped onto a silica gel plate to fabricate the colorimetric sensor. Subsequently, the CSA was placed in a fume hood for 10 min to ensure that the solvent completely evaporated. The color‐sensitive dyes used in this study are as follows:^[^ ^39^ ^]^

1. 5, 10, 15, 20‐Tetraphenyl‐21H, 23H‐porphin;
2. 5, 10, 15, 20‐Tetraphenyl‐21H, 23H‐porphine manganese (III) chloride;
3. 5, 10, 15, 20‐Tetrakis (4‐methoxyphenyl) ‐21H, 23H‐porphine iron (III) chloride;
4. 5, 10, 15, 20‐Tetraphenyl‐21H, 23H‐porphine iron (III) chloride
5. 5, 10, 15, 20‐Tetraphenyl‐21H, 23H‐porphine zinc;
6. Basic red.

### Performance verification of the Colorimetric Sensor Arrays

The required relative humidity (RH) of the gas chamber is achieved by the saturated solution and testing it with a digital humidity sensor. Sensor arrays of three variations (Dye, Dye@ZIF‐8, and Dye@ZIF‐8@COF) were positioned in chambers set to varying RH levels for a duration of 12 min. Images of the colorimetric sensor arrays before and after exposure to humidity were recorded using a 3CCD (AT‐200GE, JAI, Denmark).

A total of 180 Dye@ZIF‐8@COF sensors (15 × 4 × 3 = 180) were fabricated and stored in a vacuum‐sealed bag after drying in an oven at 50 °C for 30 min. The sensors stored at room temperature were used to detect four VOCs (ethyl acetate, hexanal, heptanal, and nonanal) daily for 15 days. Three parallel experiments were taken for each VOC detection. The stability of the sensors was assessed by observing the changes in the response values.

The prepared Dye@ZIF‐8@COF sensors were used to detect VOCs at various concentrations (1, 5, 10, 25, 50, 100, 500, and 1000 ppm for ethyl acetate, hexanal, heptanal, and nonanal). Standard curves were constructed using the sensor response values and the logarithmic values of VOCs concentrations, and the standard deviation of the sensor response values was calculated. The detection limit (LOD) was then determined using the formula LOD = 3 SD m^−1^, where SD is the standard deviation of the blank experiment (*n* = 10) and m is the slope of the standard curve.

### Detection of VOCs Released from Matcha Drying Processes Using the Sensors

The matcha drying process is notably a crucial stage for generating a substantial number of VOCs, which contribute to matcha's characteristic aroma. Moreover, this process is usually accompanied by significant changes in moisture content, which poses a challenge to sensors in terms of their humidity resistance and sensitivity. Therefore, the matcha samples from the drying process serve as ideal analytes for evaluating the sensor's performance. Transfer 2 g of sample into an aluminum box (53 cm × 57 cm) equipped with the Dye@ZIF‐8@COF colorimetric sensor arrays. Place the aluminum box with a lid at room temperature for 8 min, allowing the VOCs and the sensor array to interact. Capture the images of the sensor array before and after exposure to VOCs using a 3CCD camera for documentation.

### Model Analysis

The detection response is obtained by subtracting the red, green, and blue (R, G, B) color values of each pixel in the pre‐exposure image from the corresponding values in the post‐exposure image, achieving a total of 18 feature variables (3 color values ×6 dyes). The details are available in the Supporting Information, Experimental Section. PCA was employed to visualize the clustering potential between different matcha drying stages, with the objective of searching and removing the outliers from these characteristic variables. 140 samples were randomly sorted, and then the calibration set and prediction set were divided according to the ratio of 2:1. Discrimination models were established with 18 feature variables as the inputs and the actual drying stages as the outputs. Three classical algorithms (i.e., LDA, SVM, and CART) and AI‐assisted 1D‐CNN were used for modeling. These identification models are described in detail in the Supporting Information, Experimental Section. All these works were performed on the Matlab 2016 (Mathworks, Natick, USA).

### Design of the AI‐Assisted BOLA Algorithm

The AI‐assisted 1D‐CNN neural network architecture comprises several key layers for effective feature extraction and classification (Figure 5A). In the first layer, a 1D convolutional layer (conv1) with 32 output channels employs a kernel size of 3, a stride of 1, and padding of 1. This layer extracts local features from the input data, introducing nonlinearity through the Rectified Linear Unit (ReLU) activation function. Subsequently, a batch normalization layer (bn1) operates on the output of 32 channels, aiding in reducing internal covariate shifts and providing regularization benefits. Following this, a max pooling layer (max_pool1) with a pooling kernel size of 2 and a stride of 2 reduces the dimensionality of the data while retaining essential features. The second 1D convolutional layer (conv2) consists of 256 output channels, a kernel size of 3, a stride of 1, and padding of 1, performing additional feature extraction with ReLU activation. Batch normalization (bn2) is applied to the output of 256 channels, mirroring the structure of the earlier batch normalization layer. A subsequent max pooling layer (max_pool2) with a pooling kernel size of 2 and a stride of 2 further reduces dimensionality. The flattening layer (flatten) transforms the multidimensional data into one dimension for seamless input into the fully connected layers. To prevent overfitting, a dropout layer with a dropout rate of 0.2 randomly omits a fraction of neurons during forward propagation. Two fully connected layers (fc1 and fc2) follow, with 1024 input features and 64 output features for fc1, 64 input features, and 7 output features for fc2, representing the final classification layer. ReLU activation functions are applied after each convolutional and fully connected layer, and a softmax function normalizes the output of the last fully connected layer, providing interpretable probabilities for each class. This comprehensive architecture balances feature extraction, nonlinearity, and regularization to facilitate effective model training and classification.

The mean squared error (MSE) was employed as the loss function during the training phase of the AI‐Assisted 1D‐CNN model, which has been widely applied in regression applications.^[^ ^40^ ^]^ Moreover, the variations in MSE loss across successive epochs can be employed to assess the convergence of the model training process. The formula used for calculating MSE loss is as follows:

(1) $$\mathrm{MSE}\;\mathrm{loss}=\frac{\sum_{i=1}^{n}{\left({\mathrm{yi}}^{\prime }-\mathrm{yi}\right)}^{2}}{n}$$

where n is the number of matcha drying samples in the calibration set, yi' and yi are the predicted moisture content and reference moisture content of the matcha drying samples, respectively. The MSE loss was minimized through the gradient descent algorithm provided by the Adaptive Moment Estimation (Adam) optimizer,^[^ ^41^ ^]^ and L2 regularization was employed to prevent the model from overfitting to the training data and enhance its generalization capability.^[^ ^42^ ^]^ The specific hyperparameter settings for the training process of 1D‐CNN were determined using the Tree‐structured Parzen Estimator (TPE) optimization method. The training process is shown in Figure 5A.

### Statistical Analysis

The data set (*n* = 21) derived from HS‐SPME‐GC‐MS in triplicate samples was calculated and expressed as the mean ± standard deviation (SD). Additionally, the HS‐SPME‐GC‐MS data set was analyzed by the one‐way analysis of variance (ANOVA) to ascertain the significance of the differences among the seven stages. The statistical analysis was performed on SPSS 21 (International Business Machines Corporation, USA). A *p*‐value of <0.05 was deemed to be statistically significant. The degree of statistical significance was indicated by the use of asterisks in the figures, as follows: ^*^ *p* < 0.05, ^**^ *p* < 0.01, ^***^ *p* < 0.001.

## Conflict of Interest

The authors declare no conflict of interest.

## Supporting information



Supporting Information

## Data Availability

The data that support the findings of this study are available from the corresponding author upon reasonable request.
