# Supplementary material for: Integrating Humidity‐Resistant and Colorimetric COF‐on‐MOF Sensors with Artificial Intelligence Assisted Data Analysis for Visualization of Volatile Organic Compounds Sensing
Source: Adv Sci (Weinh). 2025 Jan 31;12(12):2411621. doi: 10.1002/advs.202411621 (PMC11947987; doi:10.1002/advs.202411621)
Supplement: Supplementary file 1 — Supporting Information [file ADVS-12-2411621-s001.docx]

**Supporting Information**

**Integrating Humidity-Resistant and Colorimetric COF-on-MOF Sensors with Artificial Intelligence Assisted Data Analysis for Visualization of Volatile Organic Compounds Sensing**

Qin Ouyang ^a,b,^[[1]](#footnote-1)^*^, Yanna Rong ^a^, Gaofan Xia ^a^, Quansheng Chen ^a^, Yujie Ma ^c,^[[2]](#footnote-2)^*^, Zhonghua Liu ^d,^[[3]](#footnote-3)^*^

^a^ School of Food and Biological Engineering, Jiangsu University, Zhenjiang 212013, P.R. China

^b^ Tea industry Research Institute, Fujian Eight Horses Tea Co., Ltd, Quanzhou 362442, P.R. China

^c^ Department of Chemistry, University of Manchester, Manchester, M13 9PL, UK

^d^ National Research Center of Engineering and Technology for Utilization of Botanical Functional Ingredients, Hunan Agricultural University, Changsha, 410128, P.R. China

**Experimental section**

***1.******Colorful difference image of the colorimetric sensor arrays***

Feature variables of the sensor images were processed as follows. Gaussian filter was first used to smooth the image, and a threshold of 0.75 was optimized to extract the color-sensitive points for completely separating the color-sensitive points from the background. Then, a diameter of about 300 pixels were averaged to obtain the red-green-blue (RGB) color values of the color-sensitive points. Next, colorful difference data was obtained by subtracting the pre-reaction image from the post-reaction image of the sensor. To visualize this difference, the feature data were normalized to [0-1] using max-min normalization, and then mapping to the 0-255 (i.e., 8-bit color). Compared with the limited 40 gray scale range of the human eye,^[1]^ the colorful difference image can clearly show the difference caused by VOCs on the sensor before and after the reaction.

***2.*** ***Determination of VOCs using HS-SPME-GC-MS***

The feature sample at each of the seven stages was tested in triplicate, totally 21 samples (3 samples × 7 stages). The VOCs in 2.0 g of each sample were enriched by HS-SPME pre-concentration procedure using a 50/30 μm Divinylbenzene/Carboxen/Polydimethylsiloxane (DVB/CAR/PDMS) fiber before analysis by a GC-MS system (MS, TQ8040, Shimadzu Corporation, Kyoto, Japan). VOCs were separated by Agilent DB-5MS capillary column (60 m × 0.25 mm inner diameter × 0.25 μm film thickness), the carrier gas was helium (purity > 99.99 %), and the flow rate was 1 mL/min. A splitless model was used with the injector temperature set at 250 ℃. The temperature of the GC column chamber was set at 50 ℃ for 5 minutes, then raised to 280 ℃ at a rate of 8 ℃/min for 6 minutes, and finally raised at 20 ℃/min to 300 ℃ for 5 minutes. The MS was in ionization mode with the ion source temperature of 230 ℃ and full scan mode with a mass scan range of 35-550 m/z.

In this study, the peak total ion currents obtained by GC-MS analysis were retrieved from the NIST library (NIST, Gaithersburg, MD, USA). The retention index (RI) of each metabolite was calculated by the n-alkane series (C7-C40). Determination of VOCs by comparing NIST mass spectrometry matches. Screening for characteristic VOCs and then quantifying them using ethyl decanoate (20 µL, 1 µg/mL). The peak area of each metabolite was compared with the peak area of the internal standard ethyl decanoate to calculate the concentration of VOCs. A text file of the original data was obtained from the HS-SPME-GC-MS analysis containing retention time and information on the mass-to-charge ratio (m/z) of the VOCs in the sample. VOCs with > 80% similarity were selected for subsequent analysis.

The data was subjected to the SIMCA V14.1 (Umetrics, Umea, Sweden) for multivariate statistical analysis models such as orthogonal projections to latent structures discriminant analysis (OPLS-DA) and hierarchical clustering analysis (HCA). The OPLS-DA model is generated according to a predictive component and an orthogonal component. The model is evaluated with respect to the "goodness of fit" and "predictive goodness" measured in the parameters R2Y and Q2Y. R2Y and Q2Y are obtained from 7-fold cross-validation and validated by alignment tests. The variable impact of OPLS-DA on the variable of importance (VIP) values was also calculated. VOCs with VIP values > 1.0 and *p*-values < 0.05 were used as characteristic volatiles and analyzed by principal component analysis (PCA), and correlation analysis to derive differential VOCs in the matcha drying process. The correlation analysis was conducted on Origin 2021 (OriginLab, Northampton, USA). Furthermore, PCA were used to analyze metabolic changes in a sample of drying final VOCs, and performed by Matlab 2016b (Mathworks, Natick, USA).

***3. Classical multivariate analysis***

***PCA*** is a linear, unsupervised classification method. The purpose of this analysis involves approximating statistical variables with a limited number of possible linear combinations (so-called principal components) to structure, simplify and account for large data sets.^[2]^ PCA is use linear transformation to map an n-dimensional feature to a k-dimensional space (k < n); the resulting k-dimension is a new orthogonal feature, and the k-dimensional feature is called the principal component (PC). PCi represents the ith principal component. The proportion of PCi variance to the total variance is called the contribution rate. A higher value of the contribution rate indicates a stronger ability to synthesize the information of the PCi. Analysis of the first ten principal component eigenvalues selected by PCA due to the cumulative contribution rate was 99.64% in this study.

***LDA*** is a technique commonly used for supervised pattern recognition. The ratio of maximizing inter-class distance to intra-class distance for maximum discrimination.^[3]^ The PCA method was used in this study to reduce the dimensionality of the spectrum data and use less variable information to represent the original spectrum information. In this study, PCA and LDA were combined. The PCA method reduces the dimensionality of multidimensional variables and solves the problem of the small sample set of the LDA algorithm.

***SVM*** is regarded as a powerful and rigorous mechanism acquisition model. The SVM attempts to separate the approximated samples (support vectors) into two categories using maximized margins to identify the optimal hyperplane.^[4]^ Moreover, the penalty coefficient *c* and the radial basis kernel function *g* are crucial parameters affecting the performance of the SVM model. This study only showed the optimized log_2_*_C_* at [−8, 8] and log_2_*g* at [−8, 8] to show the contour plots more clearly cross-validation accuracy of five-fold cross-validations indicates the presence of the ideal pairing of c and g. The SVM optimization model was obtained analytically using different numbers of PCs (from 1 to 10) as input variables.

***CART*** is a supervised form of hierarchical clustering classifying non-parametric data in the form of a spanning binomial tree. The probability distribution of the prediction is determined by the recursive partitioning of the independent variable regions.^[5]^ The contents of the analyzed elements in 140 samples constituted the root of the binary tree. As a result of constructing the tree, the number of splits (n=7) and the number of terminal nodes (n=5) were obtained. A detailed analysis of the split attributes for these samples is presented in **Figure S24**G. In this study, the best explanatory variables are chosen using principal component values to determine the best dichotomous cut-off point of 205.406. The models have been pruned with automatic thresholding for optimal binary trees to avoid tree overfitting.

**
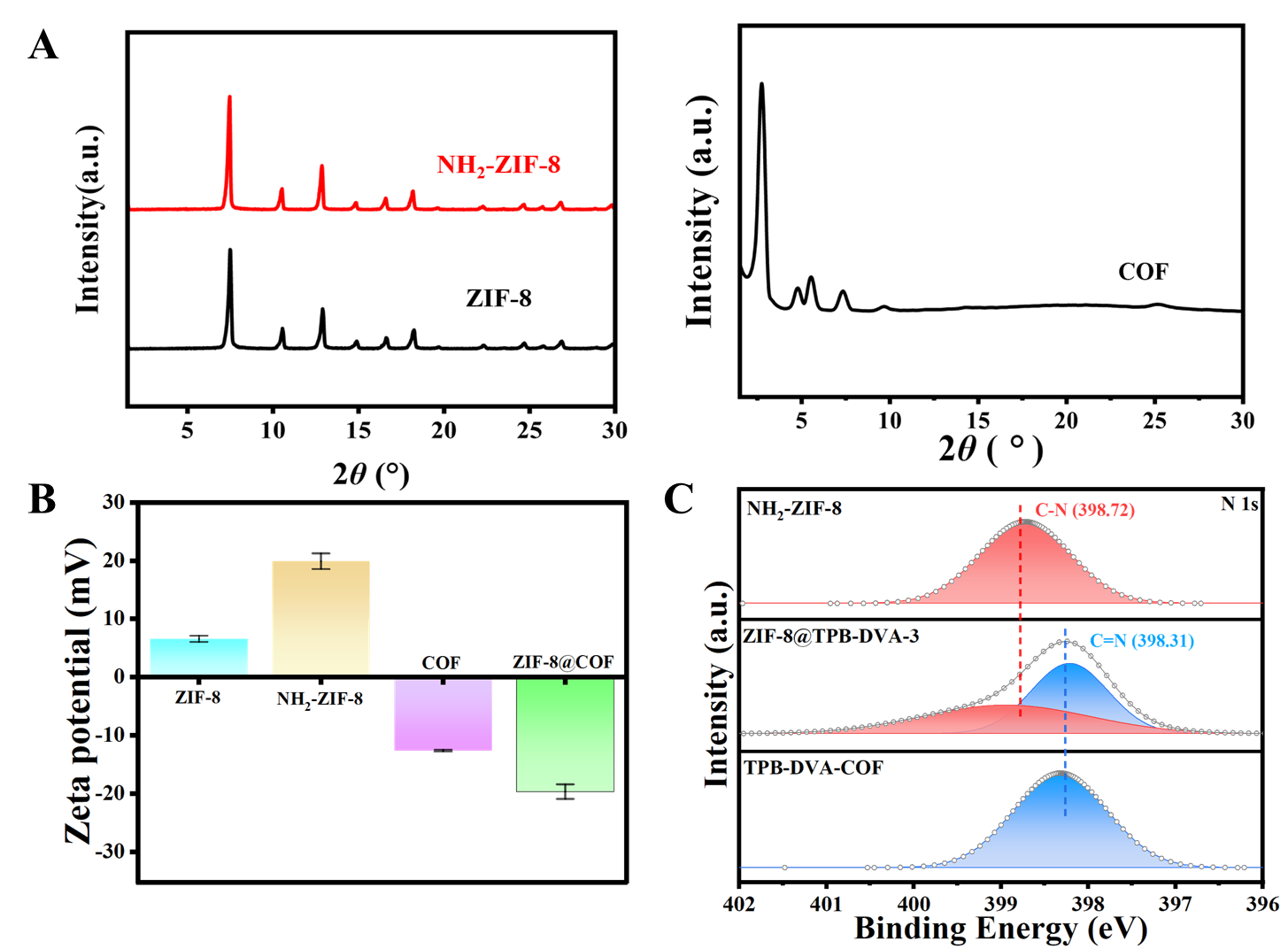
**

**Figure S1.** A) XRD patterns of ZIF-8 and COF materials. B) Zeta potential. C) N 1s XPS spectra of the materials.


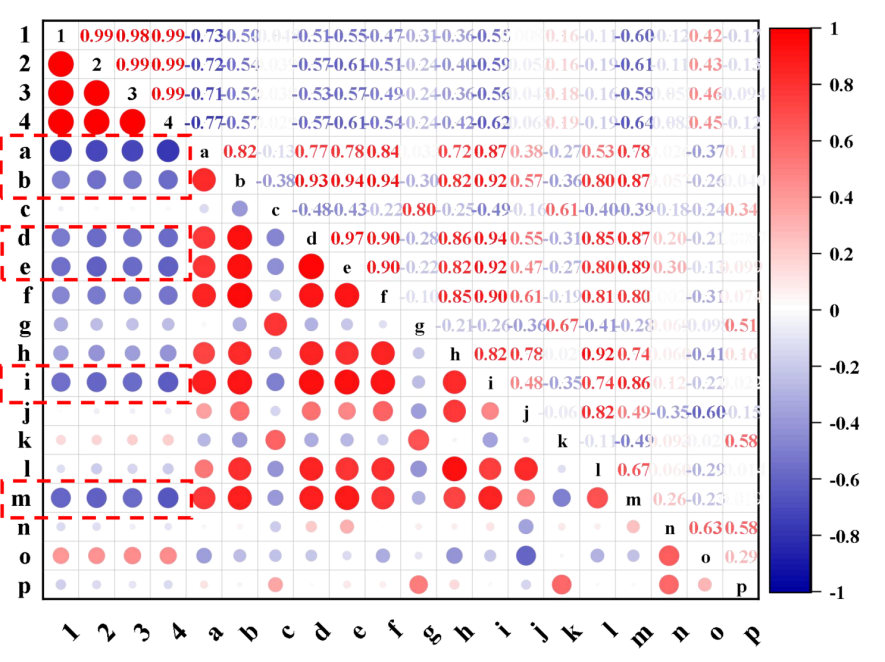


**Figure S2.** Characteristics of correlation between volatile organic compounds (VOCs) and color sensitive dyes. (a, 5, 10, 15, 20-Tetraphenyl-21H, 23H-porphin; b, 5, 10, 15, 20-Tetraphenyl-21H, 23H-porphine manganese (III) chloride; c, 2, 3, 7, 8, 12, 13, 17, 18-Octaethyl-21H, 23H-porphine manganese (III) chloride; d, Bromocresol green; e, 5, 10, 15, 20-Tetrakis (4-methoxyphenyl)-21H, 23H-porphine iron (III) chloride; f, 5, 10, 15, 20-Tetraphenyl-21H, 23H-porphine iron (III) chloride; g, 5, 10, 15, 20-Tetraphenyl-21H, 23H-porphine copper (II); h, Bromophenol blue; i, 5, 10, 15, 20-Tetrakis (pentafluorophenyl)-21H, 23H-porphyrin iron(III) chloride; j, 5, 10, 15, 20-Tetrakis (4-methoxyhenyl)-21H, 23H-porphine cobalt(II); k, 5, 10, 15, 20-Tetraphenyl-21H, 23H-porphine zinc; l, Bromothymol blue; m, 5, 10, 15, 20-Tetrakis (4-methoxyphenyl)-21H, 23H-porphine; n, 2, 3, 7, 8, 12, 13, 17, 18-Octaethyl-21H, 23H-porphine nickel (II); o, Meso-Tetraphenylporphyrin; p, Basic red.)


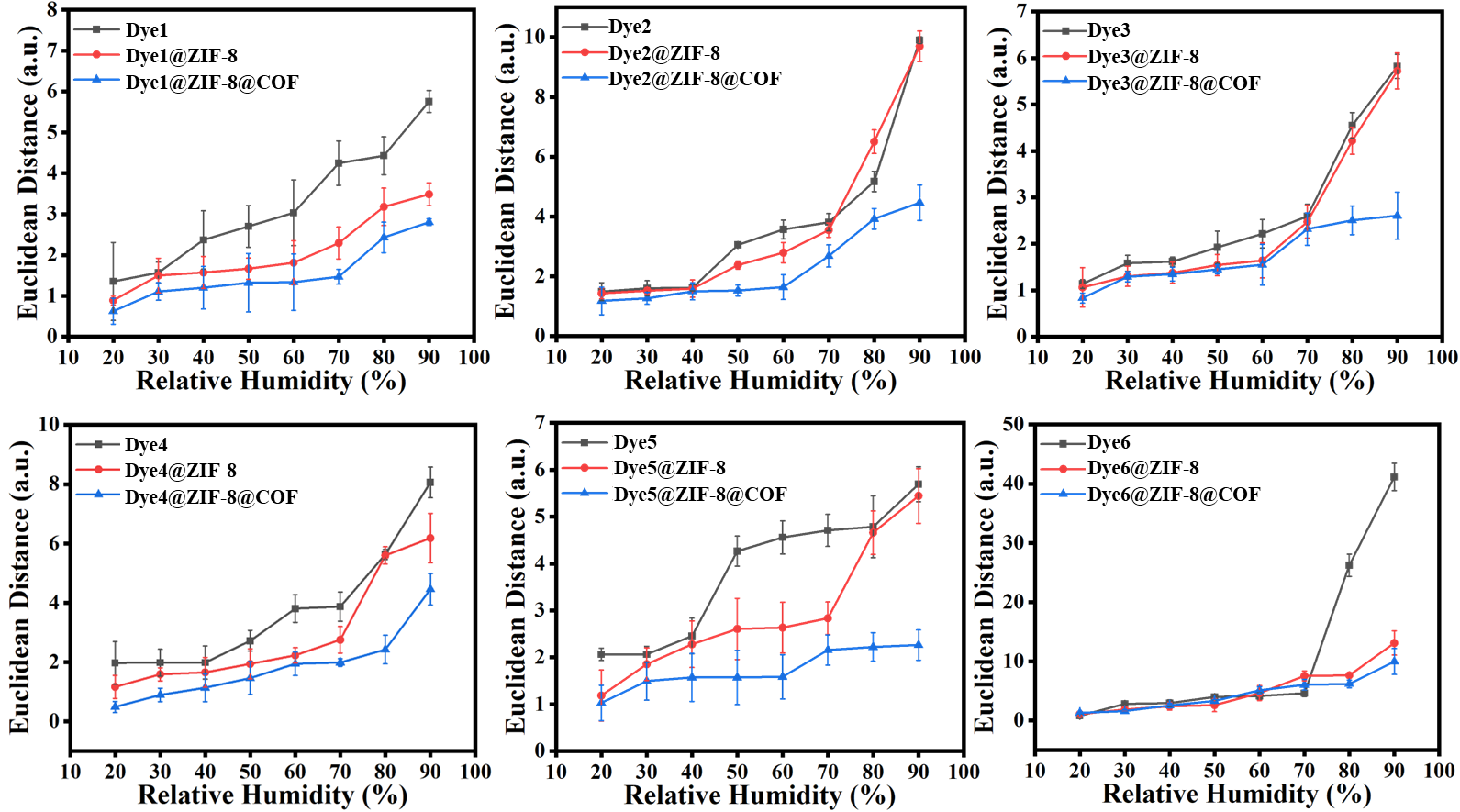


**Figure S3.** The humidity response testing of dye, Dye@ZIF-8, and Dye@ZIF-8@COF showing in ED values. Dye1, Dye2, Dye3, Dye4, Dye5, and Dye6 are listed in Table S1.


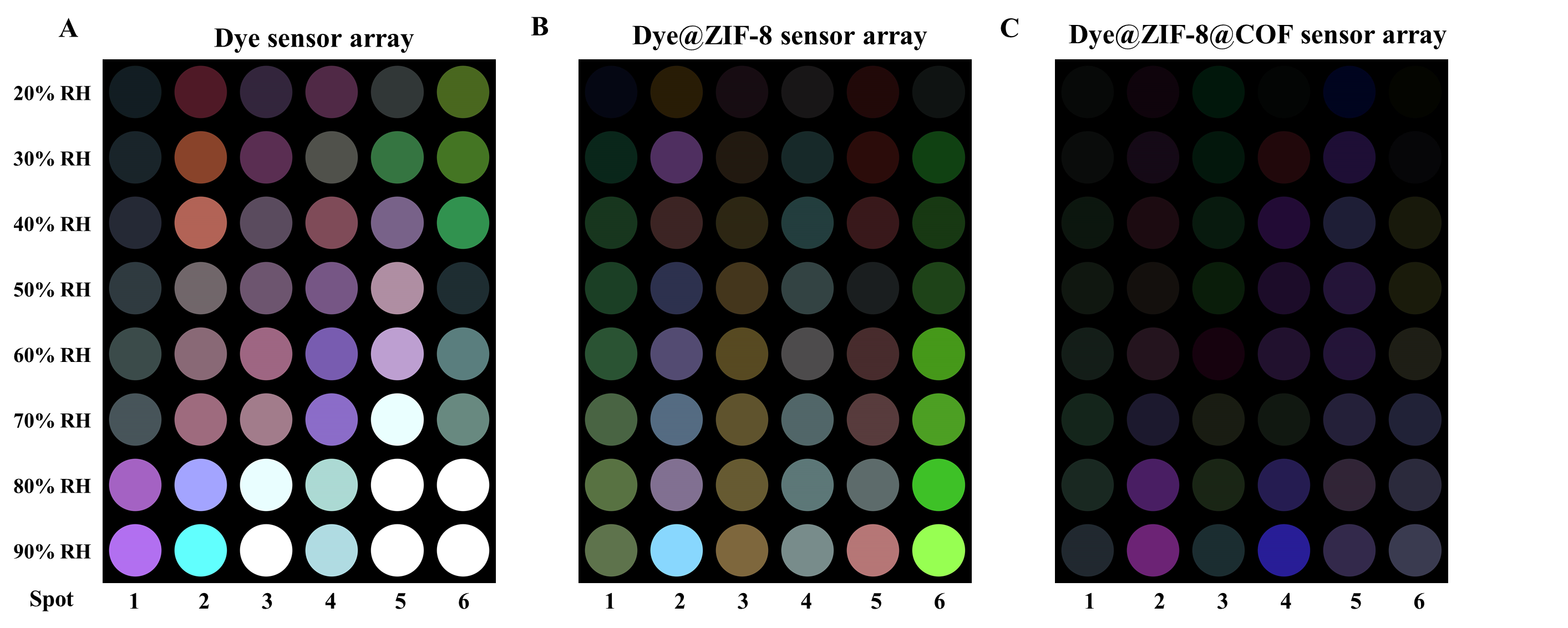


**Figure S4.** The humidity response testing of Dye, Dye@ZIF-8, and Dye@ZIF-8@COF showing in color differential profiles (A: Dye; B: Dye@ZIF-8; C: Dye@ZIF-8@COF; Spot 1-6 represent the Dye@ZIF-8@COF sensors incorporating 6 different dyes listed in Table S1).

**
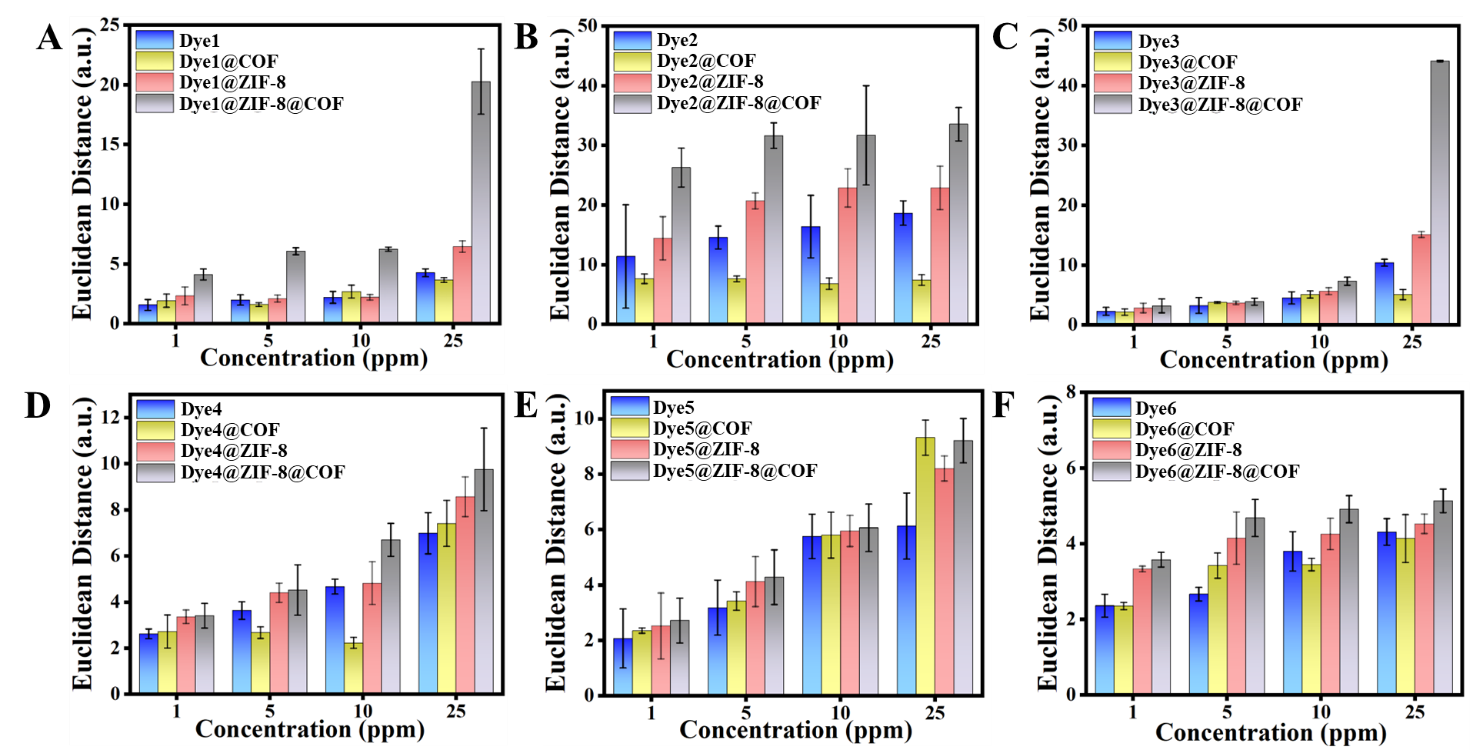
**

**Figure S5.** ED values of dye, Dye@ZIF-8, and Dye@ZIF-8@COF to ethyl acetate (n=3) with different concentrations. Dye1, Dye2, Dye3, Dye4, Dye5, and Dye6 are listed in Table S1.


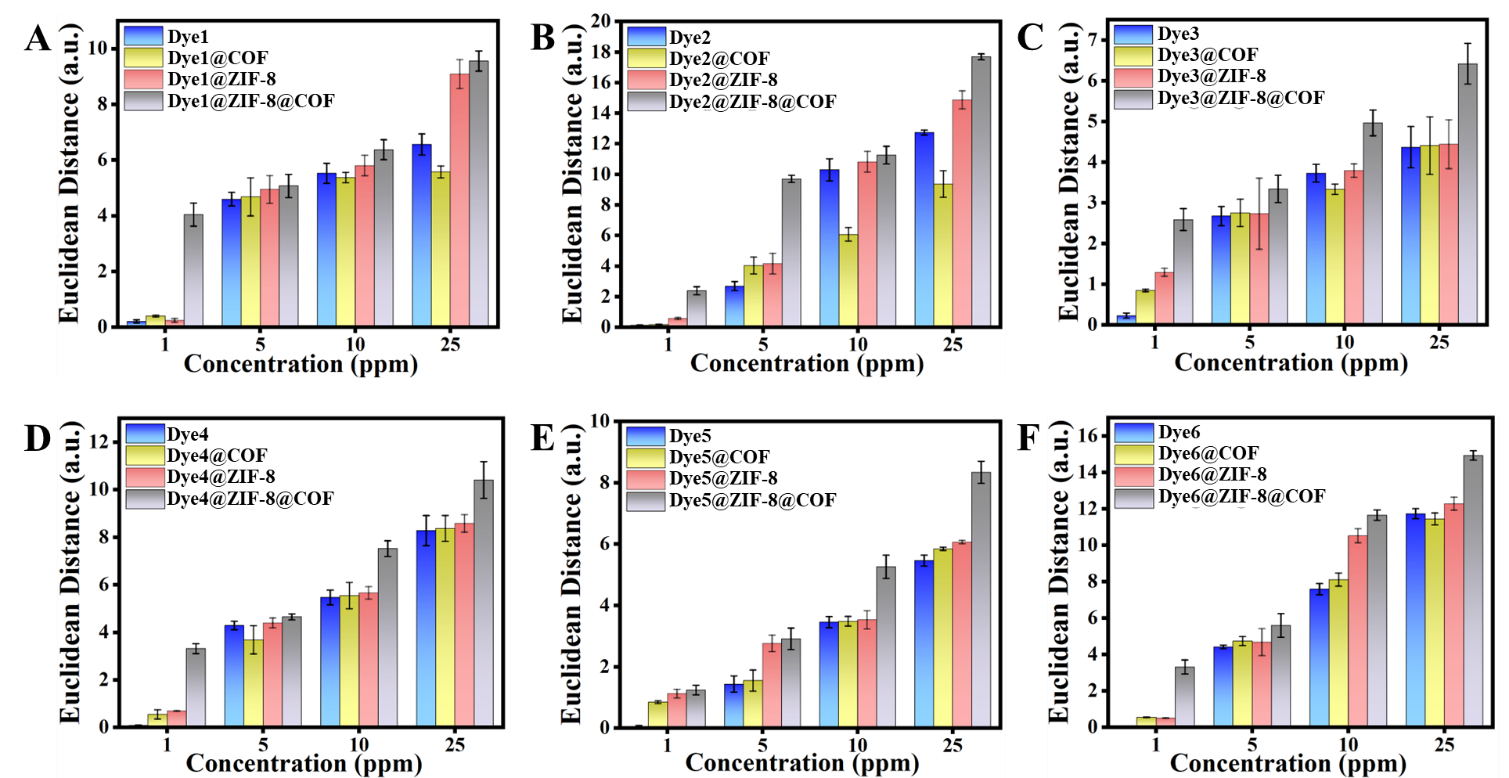


**Figure S6.** ED values of Dye, Dye@ZIF-8, and Dye@ZIF-8@COF to hexanal (n=3) with different concentrations. Dye1, Dye2, Dye3, Dye4, Dye5, and Dye6 are listed in Table S1.


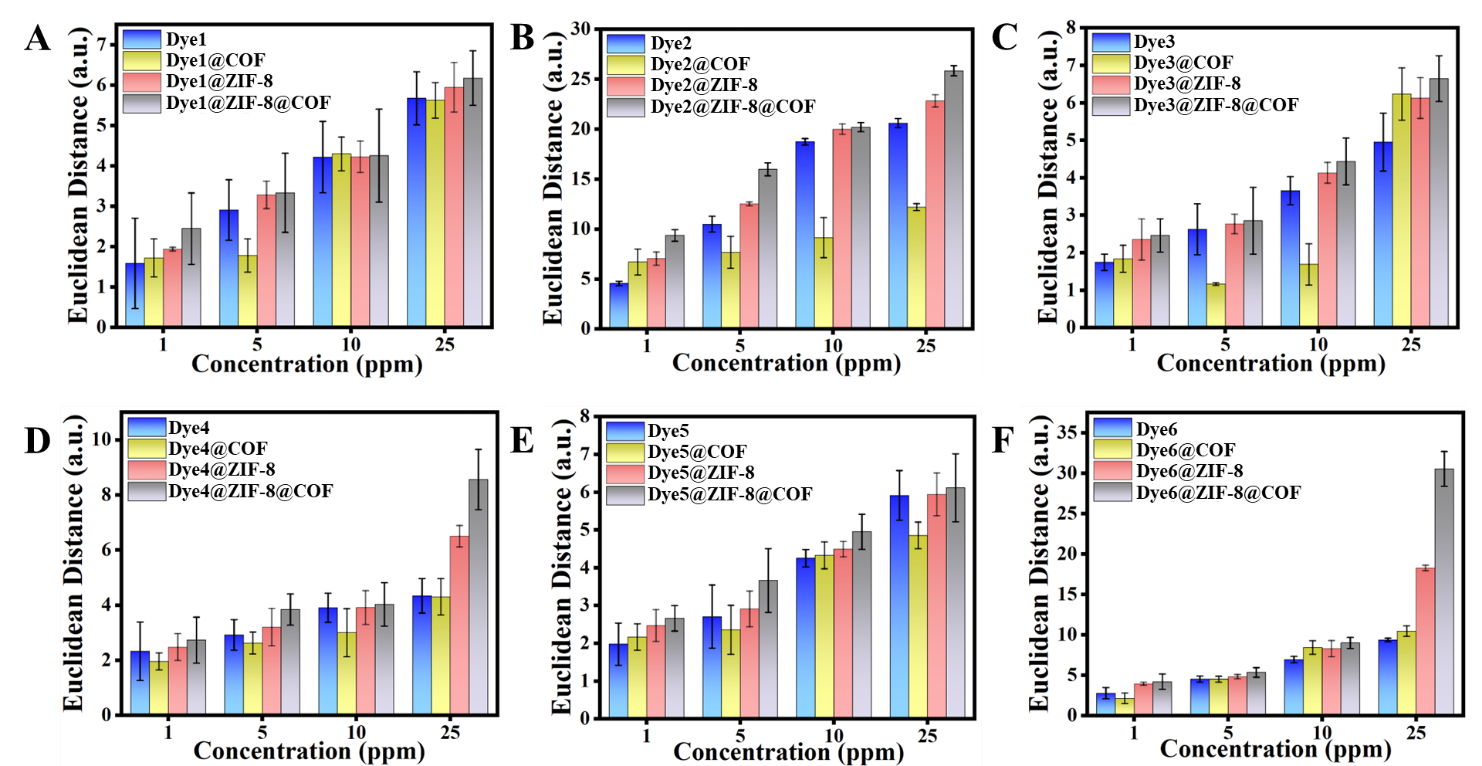


**Figure S7.** ED values of Dye, Dye@ZIF-8, and Dye@ZIF-8@COF to heptanal (n=3) with different concentrations. Dye1, Dye2, Dye3, Dye4, Dye5, and Dye6 are listed in Table S1.

**
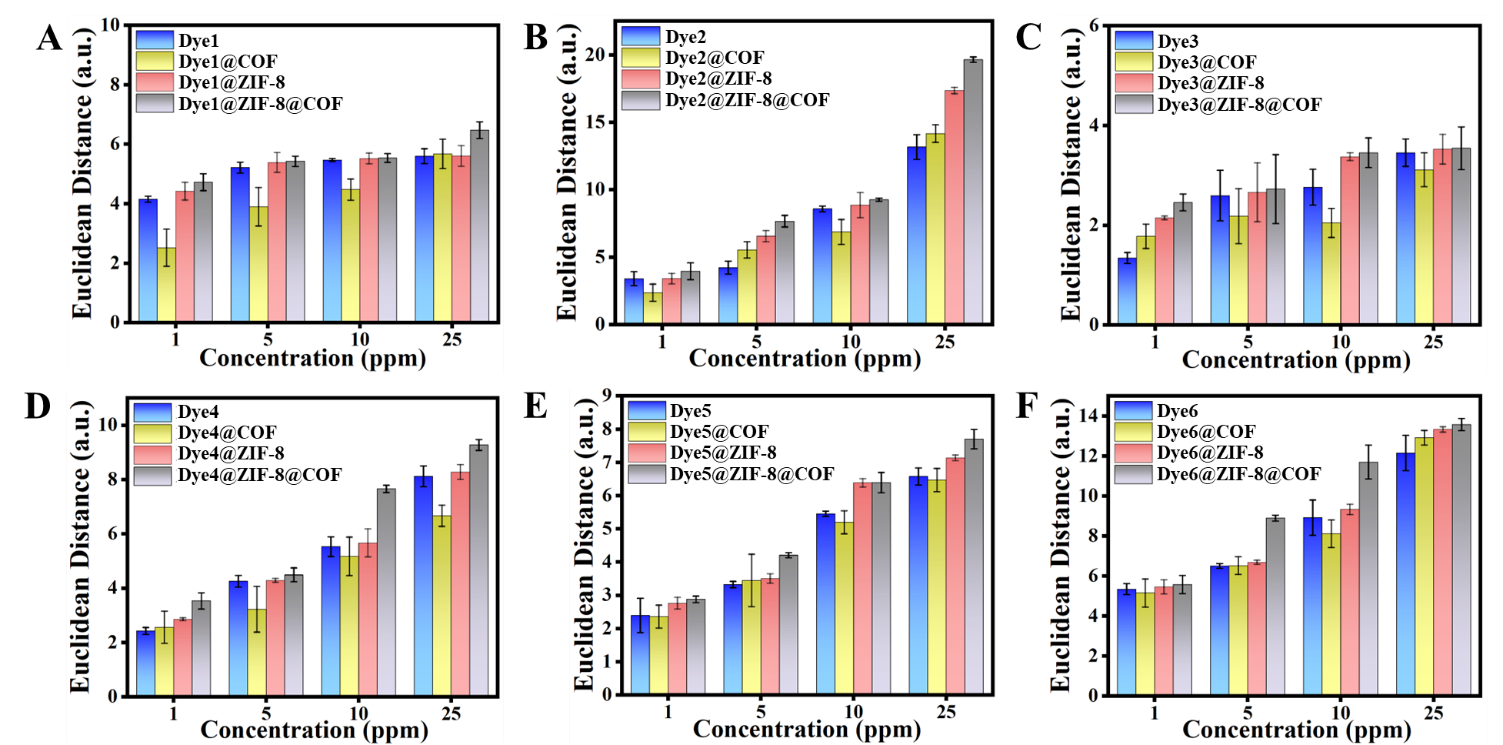
**

**Figure S8.** ED values of Dye, Dye@ZIF-8, and Dye@ZIF-8@COF to nonanal (n=3) with different concentrations. Dye1, Dye2, Dye3, Dye4, Dye5, and Dye6 are listed in Table S1.


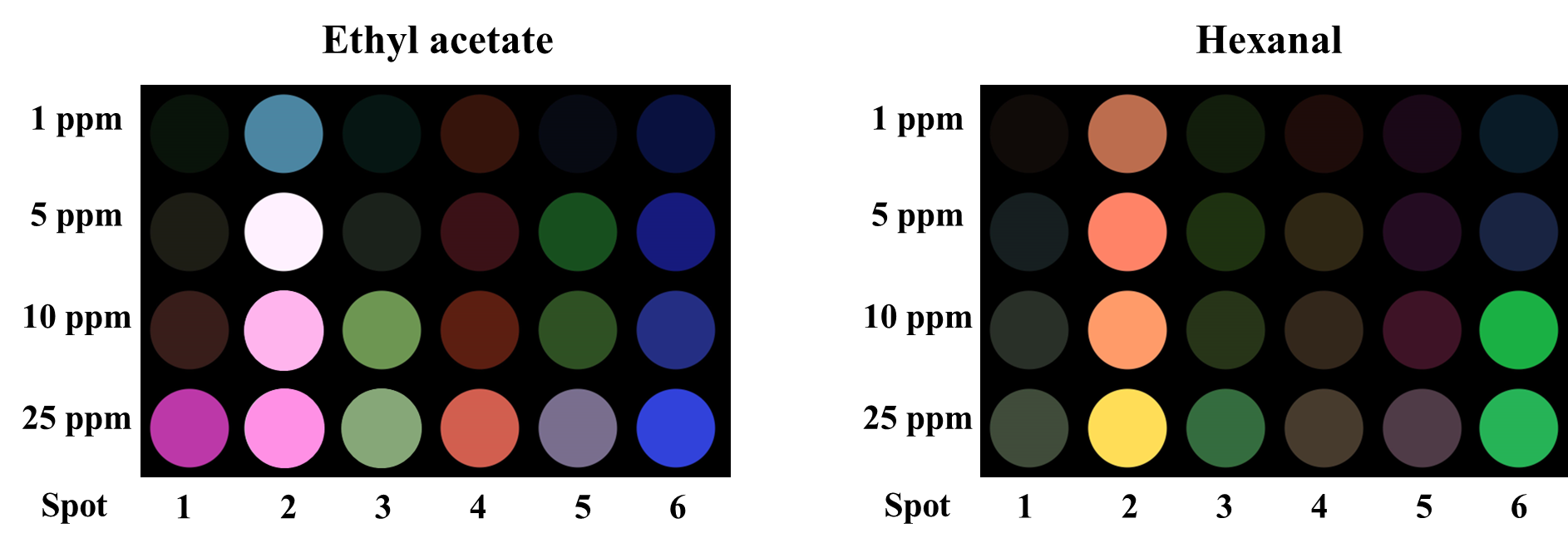


**Figure S9.** Colorful difference images of the Dye@ZIF-8@COF sensors for identification and direct visualization of VOCs (Ethyl acetate and Hexanal) sensing at different concentrations of 1-25 ppm (Spot 1-6 represent the Dye@ZIF-8@COF sensors incorporating 6 different dyes listed in Table S1).


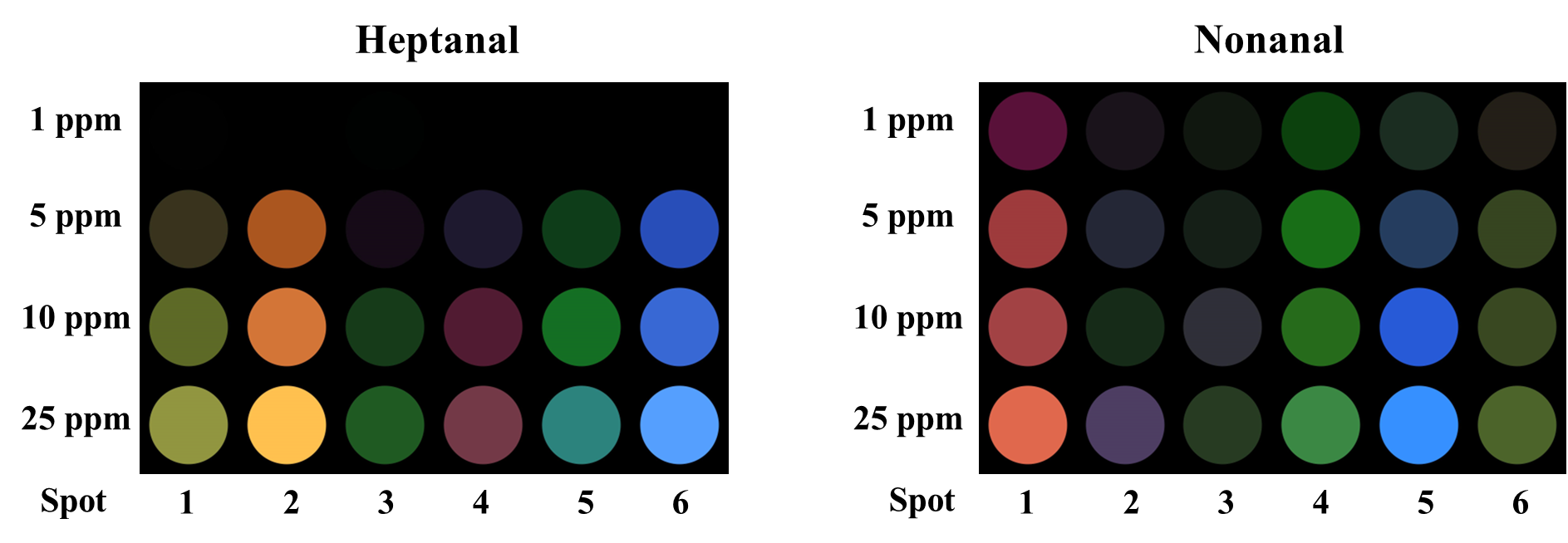


**Figure S10.** Colorful difference images of the Dye@ZIF-8@COF sensors for identification and direct visualization of VOCs (Heptanal and Nonanal) sensing at different concentrations of 1-25 ppm (Spot 1-6 represent the Dye@ZIF-8@COF sensors incorporating 6 different dyes listed in Table S1).


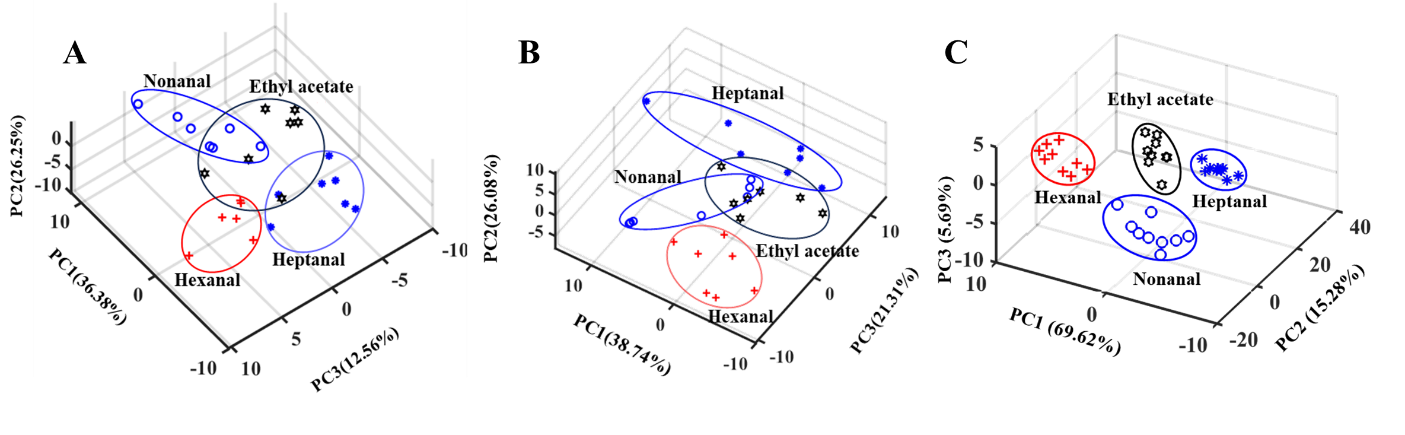


**Figure S11.** Principal components analysis of four VOC based on colorimetric sensors. A) Dye, B) Dye@ZIF-8, C) Dye@ZIF-8@COF.***
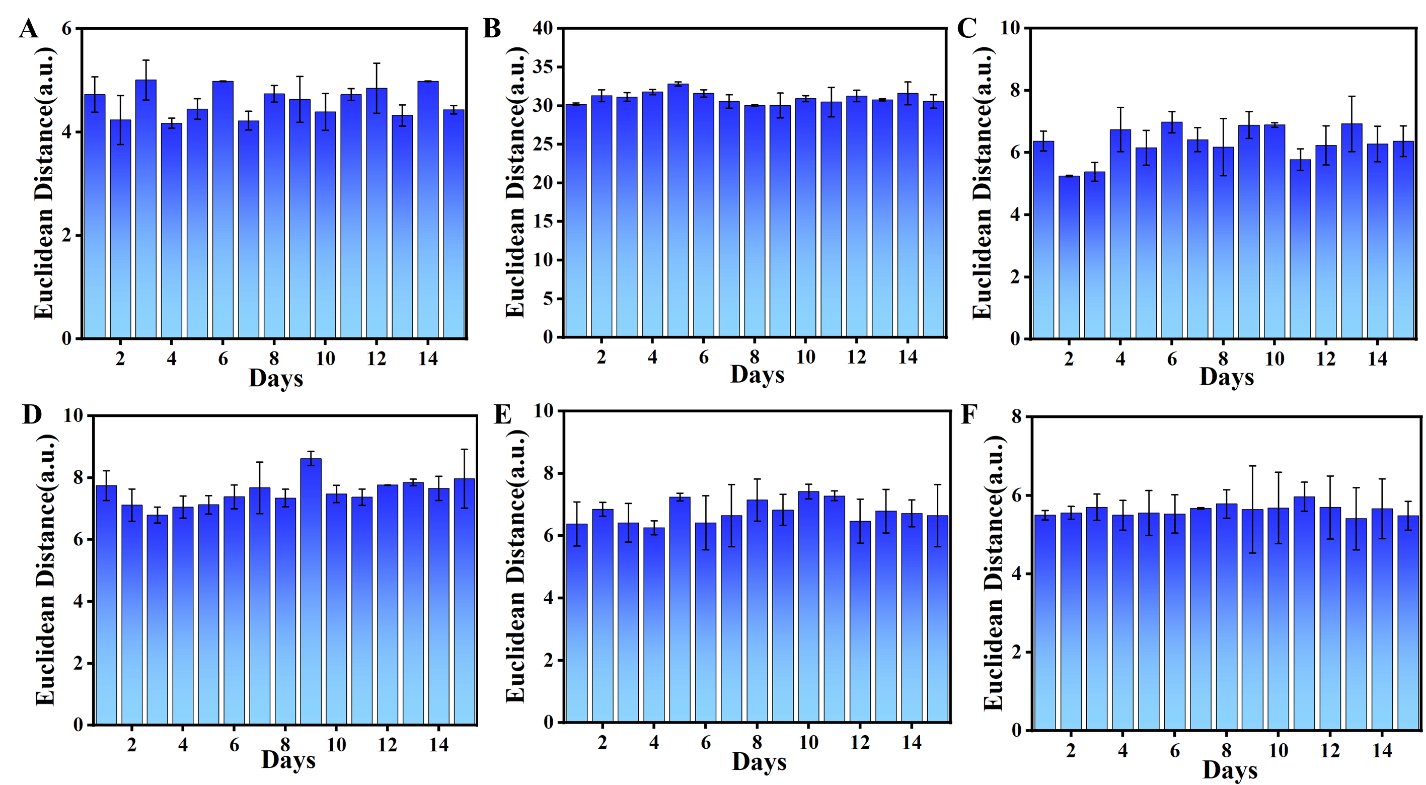
***

**Figure S12.** Stability test of Dye@ZIF-8@COF sensor for ethyl acetate sensing. A) Dye1, B) Dye2, C) Dye3, D) Dye4, E) Dye5 and F) Dye6. The six different dyes are listed in Table S1.


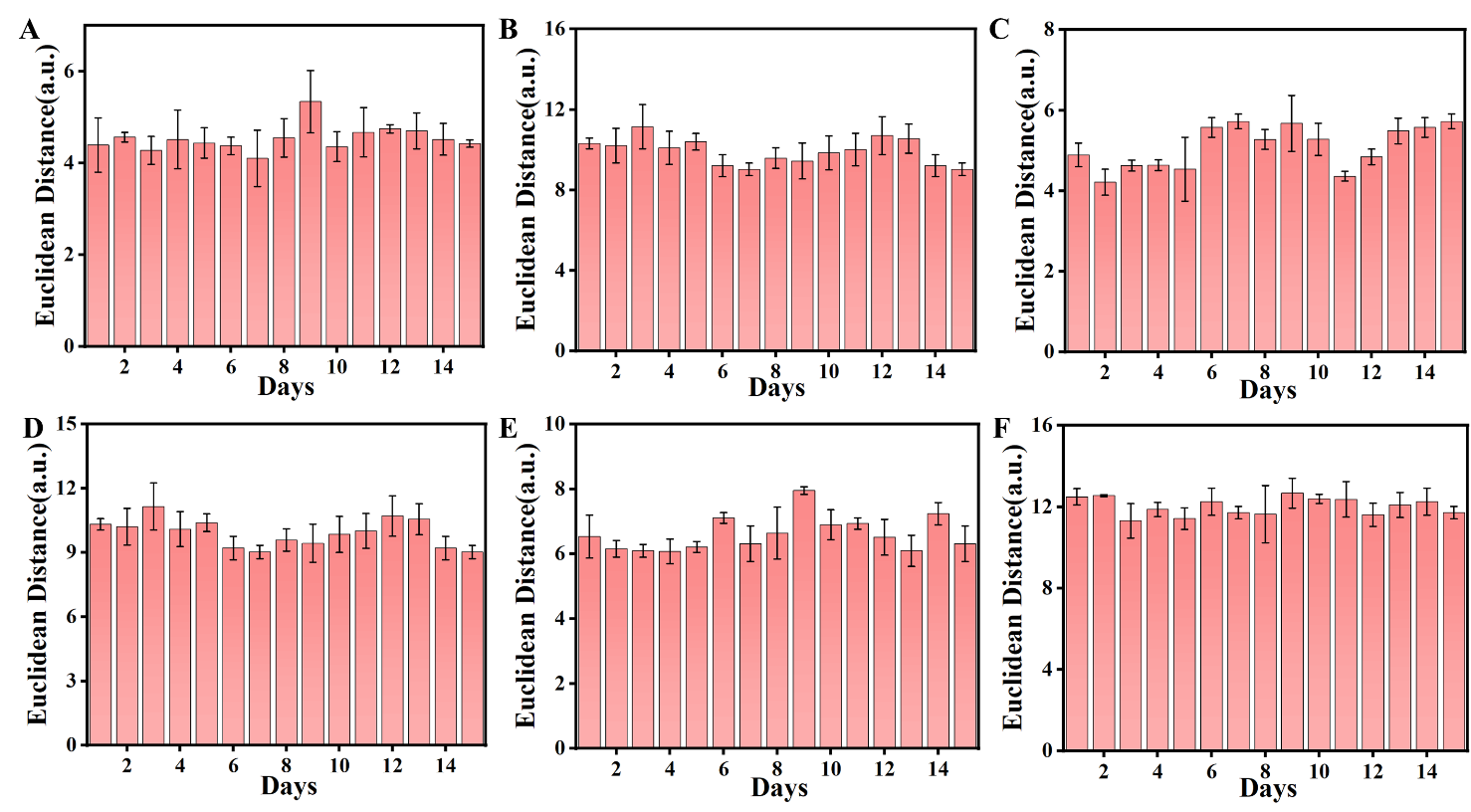


**Figure S13.** Stability test of Dye@ZIF-8@COF sensor for hexanal sensing. A) Dye1, B) Dye2, C) Dye3, D) Dye4, E) Dye5 and F) Dye6. The six different dyes are listed in Table S1.


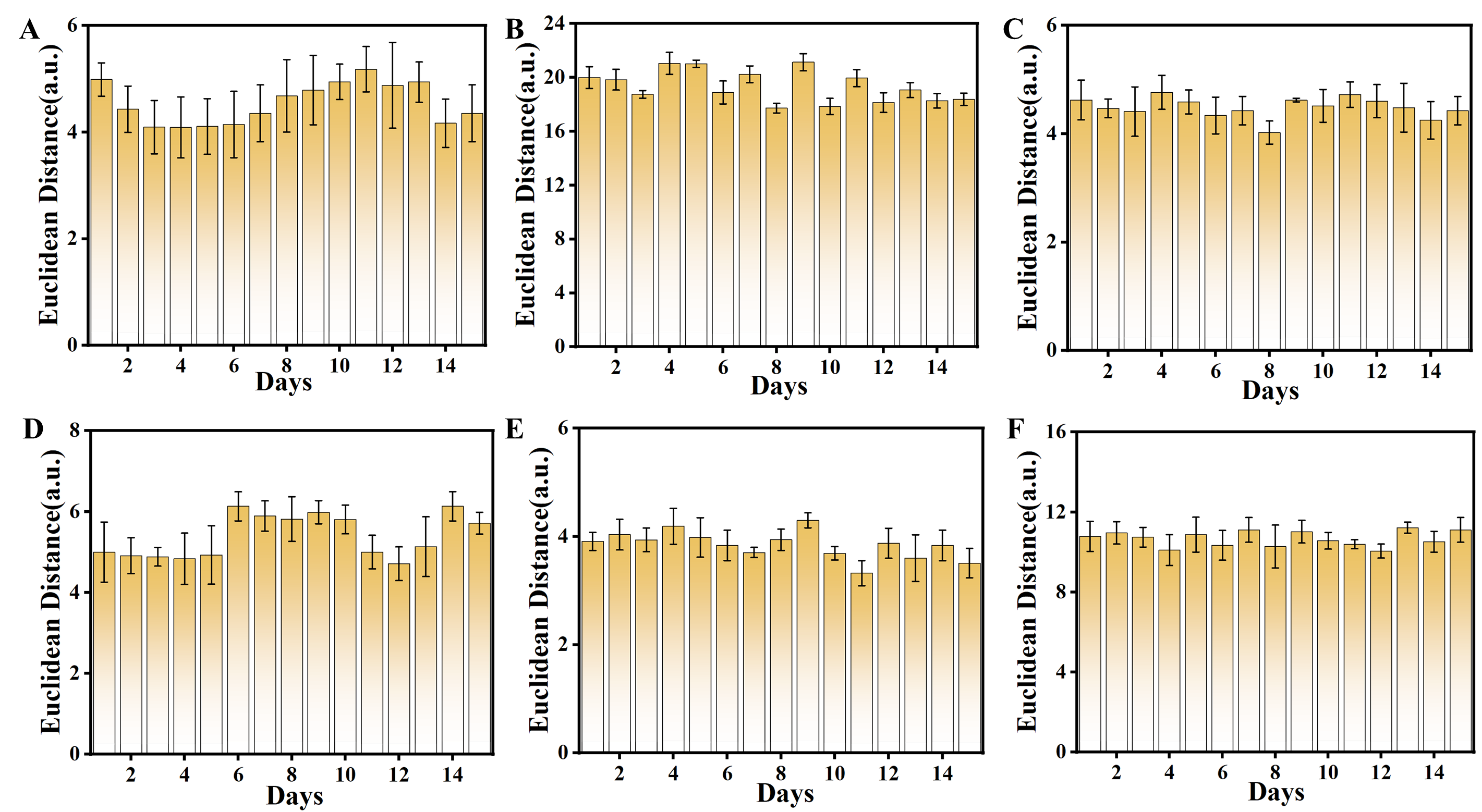
**Figure S14.** Stability test of Dye@ZIF-8@COF sensor for heptanal sensing. A) Dye1, B) Dye2, C) Dye3, D) Dye4, E) Dye5 and F) Dye6. The six different dyes are listed in Table S1.


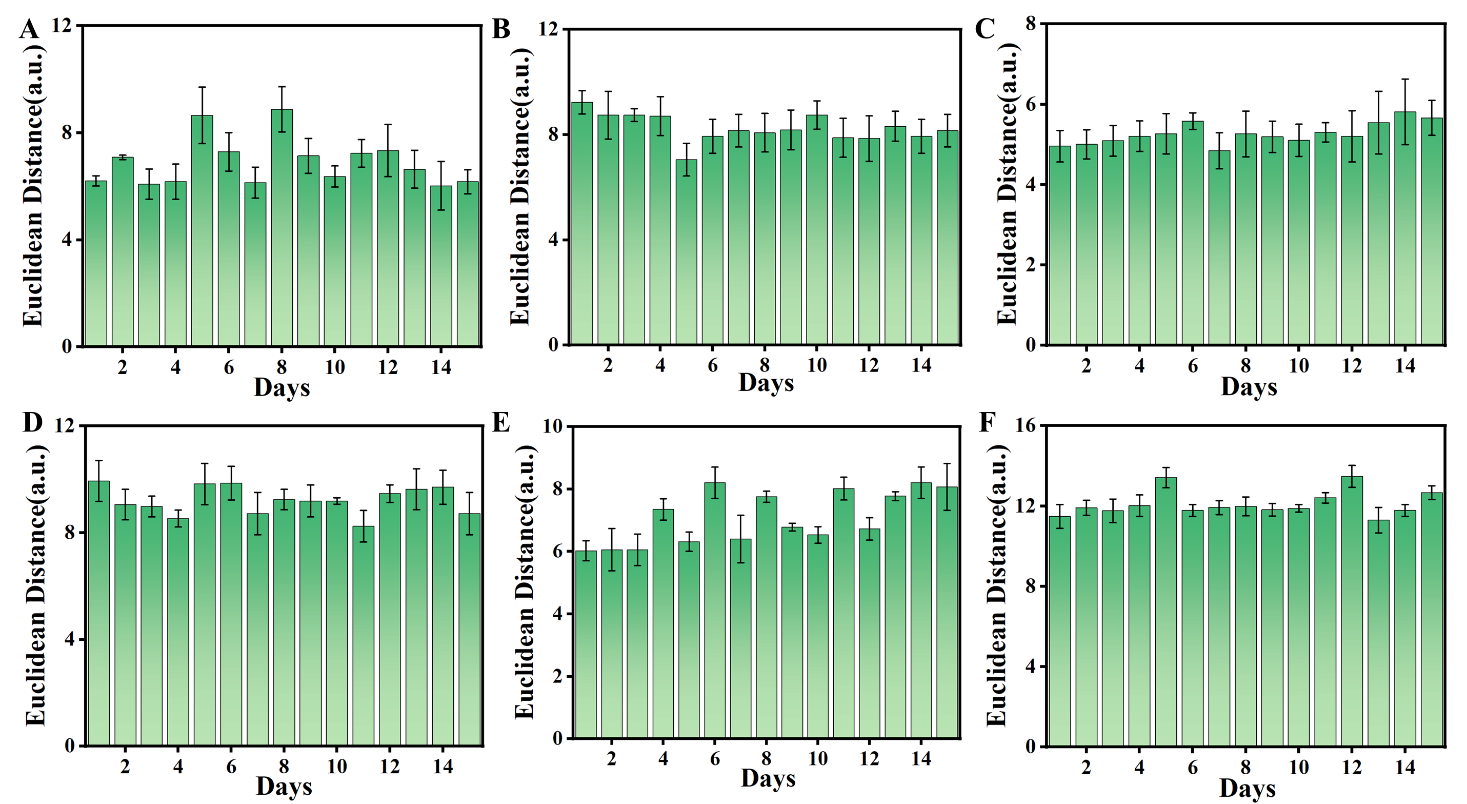


**Figure S15.** Stability test of Dye@ZIF-8@COF sensor for nonanal sensing. A) Dye1, B) Dye2, C) Dye3, D) Dye4, E) Dye5 and F) Dye6. The six different dyes are listed in Table S1.


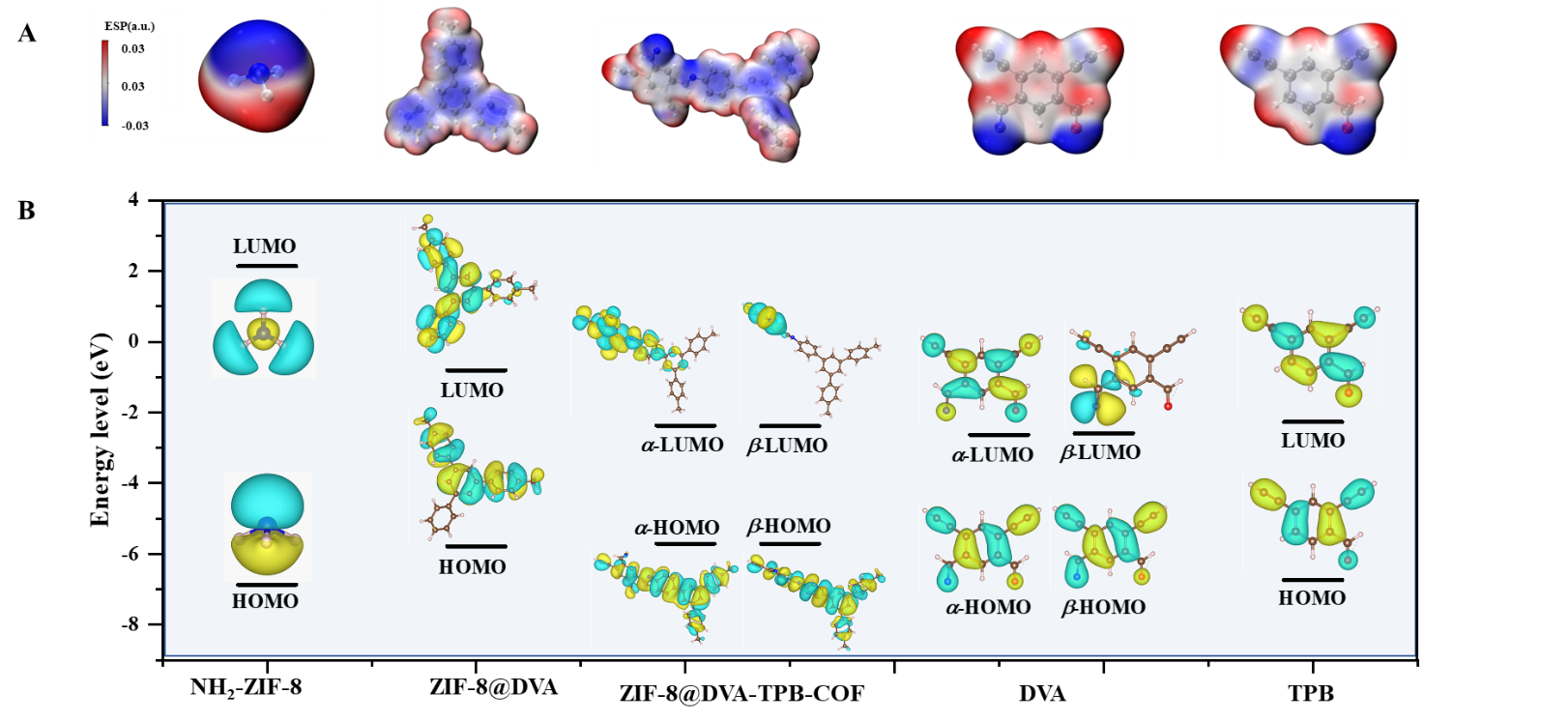


**Figure S16.** A) Electrostatic potentials of NH_2_-ZIF-8, ZIF-8@DVA, ZIF-8@DVA-TPB-COF, DVA and TPB. B) Simulation results of the HOMO and LUMO energy levels of NH_2_-ZIF-8, ZIF-8@DVA, ZIF-8@DVA-TPB-COF, DVA and TPB.


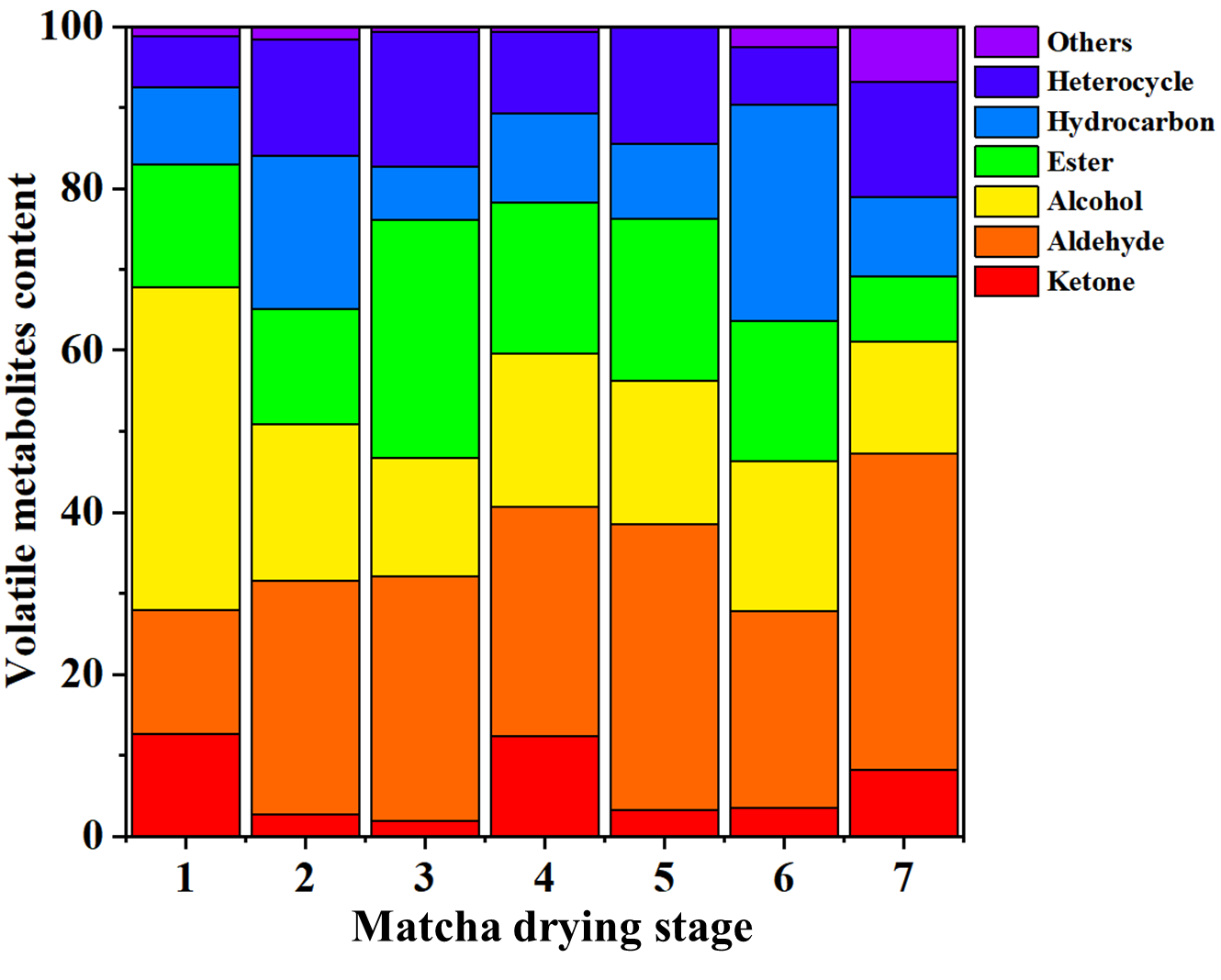


**Figure S17.** VOCs content of matcha drying process analyzed by HS-SPME-GC-MS.


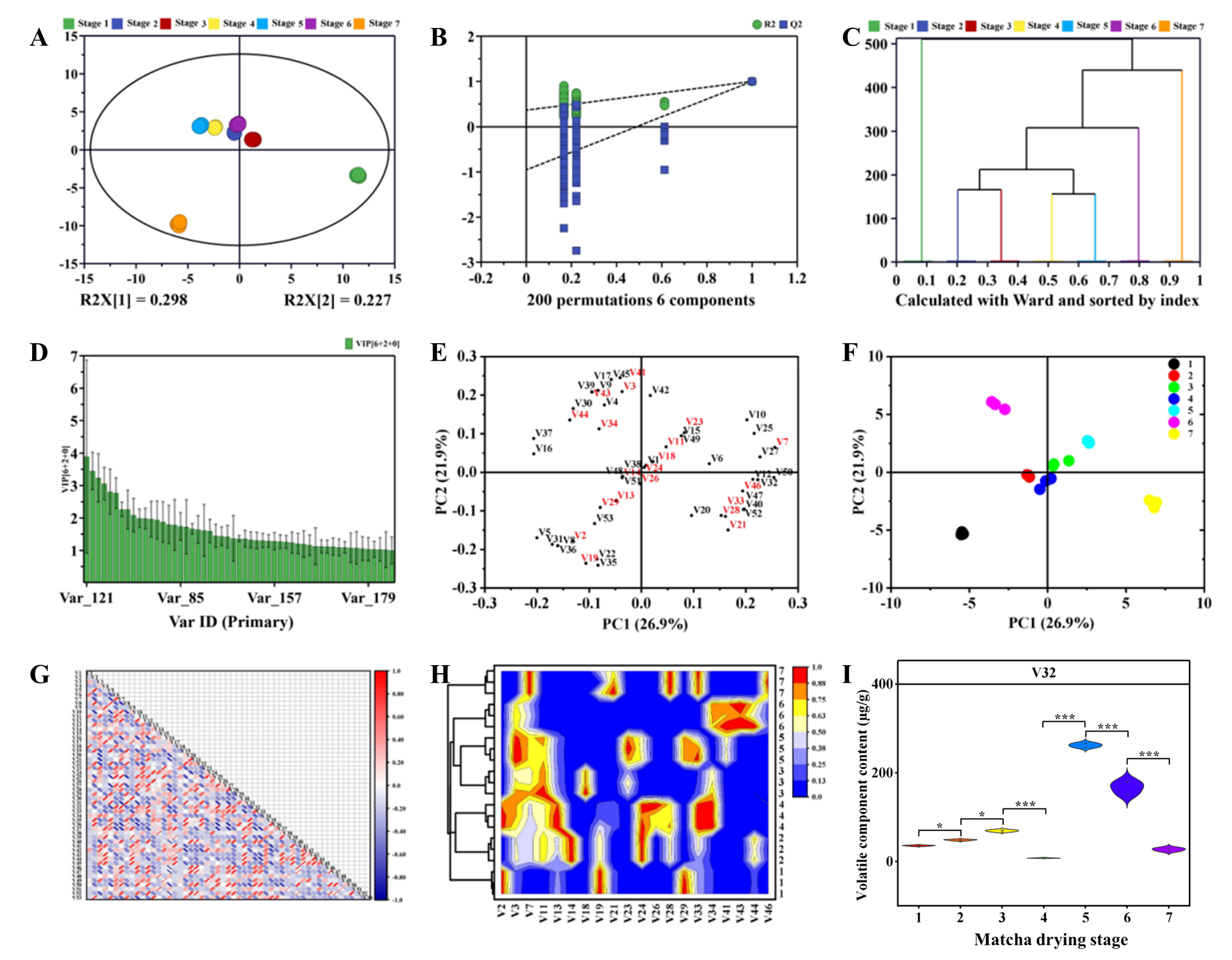


**Figure S18.** Statistical analysis of the content of VOCs during matcha drying process. A) Orthogonal projections to latent structures discriminant analysis (OPLS-DA) score; B) its permutation test plot (200 permutation tests) for samples; C) cluster analysis; D) VIP values sorted with OPLS-DA; E) score plot of VOCs with VIP values > 1.0; F) loading plot of VOCs with VIP values > 1.0; G) Pearson correlation plot of the VOCs with VIP values > 1.0; H) heat map of the steady signal of characteristic VOCs and I) characteristic VOCs (Nonanal) during matcha drying process analyzed by ANOVA, **p* < 0.05, ***p* < 0.01, ****p* < 0.001.


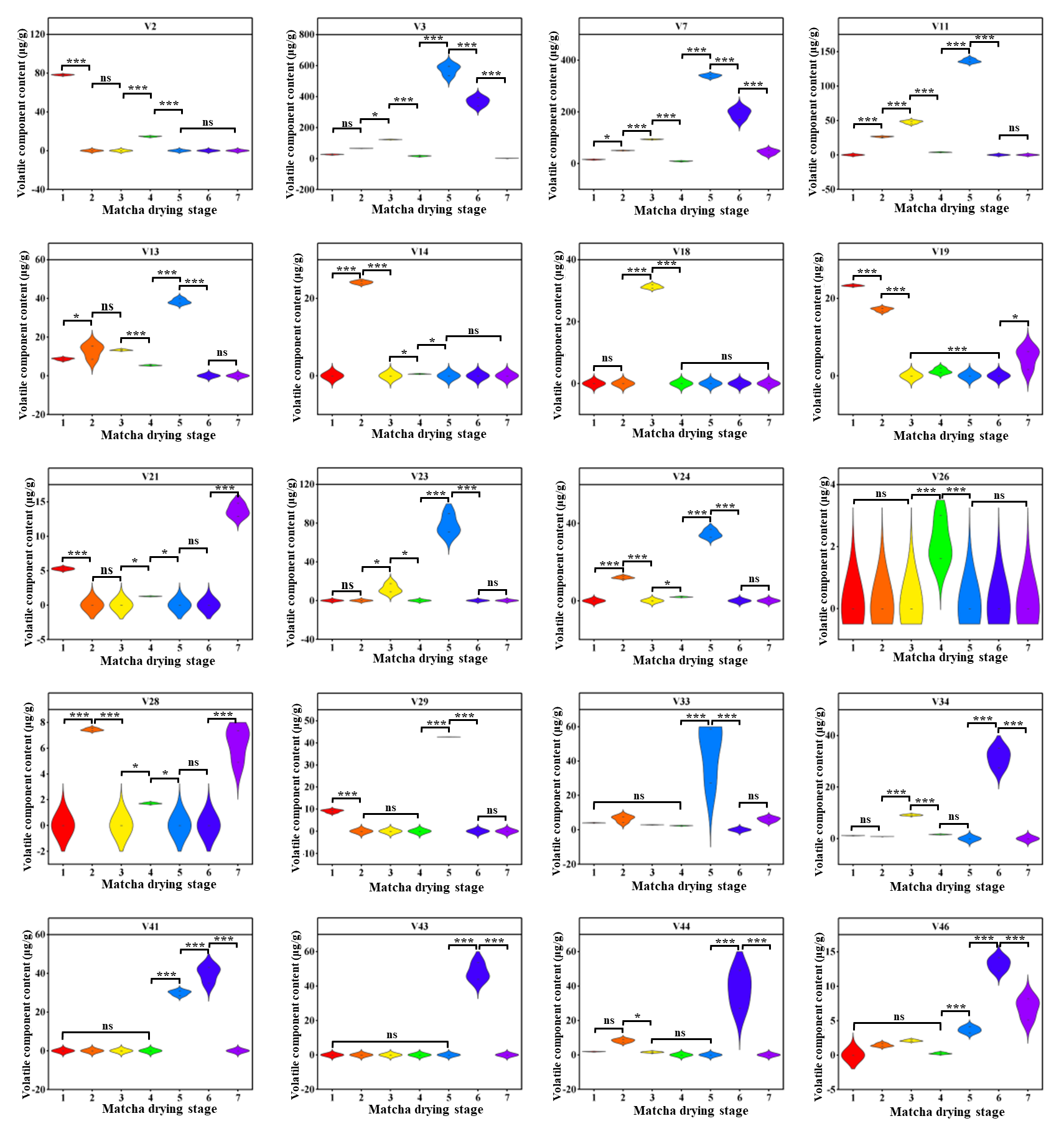


**Figure S19.** The violin figures of VOCs showing significant differences in matcha drying process, **p* < 0.05, ***p* < 0.01, ****p* < 0.001.


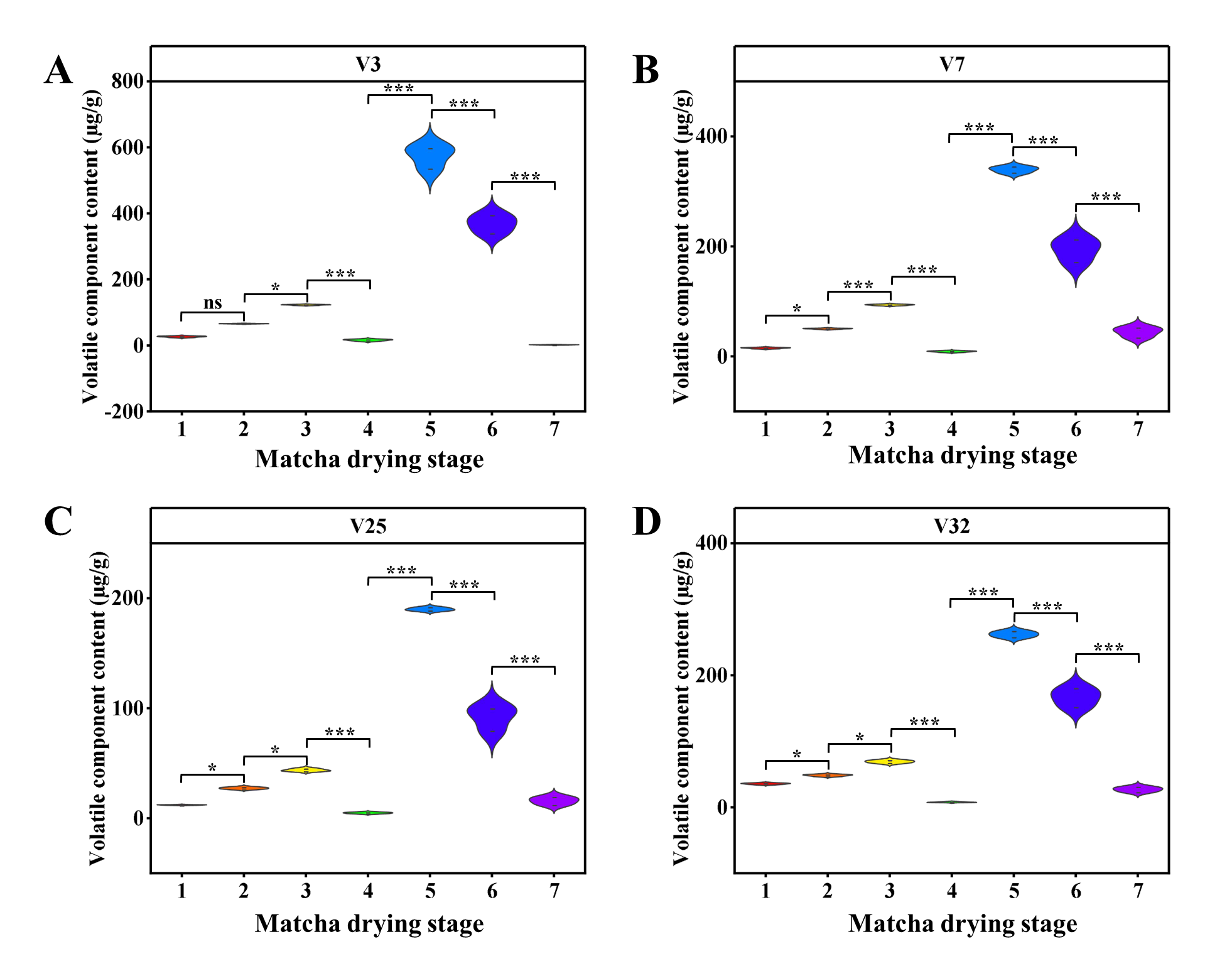


**Figure S20.** The violin figures of ethyl acetate (V3), hexanal (V7), heptanal (V25) and nonanal (V32) showing significant differences in matcha drying process, **p* < 0.05, ***p* < 0.01, ****p* < 0.001.


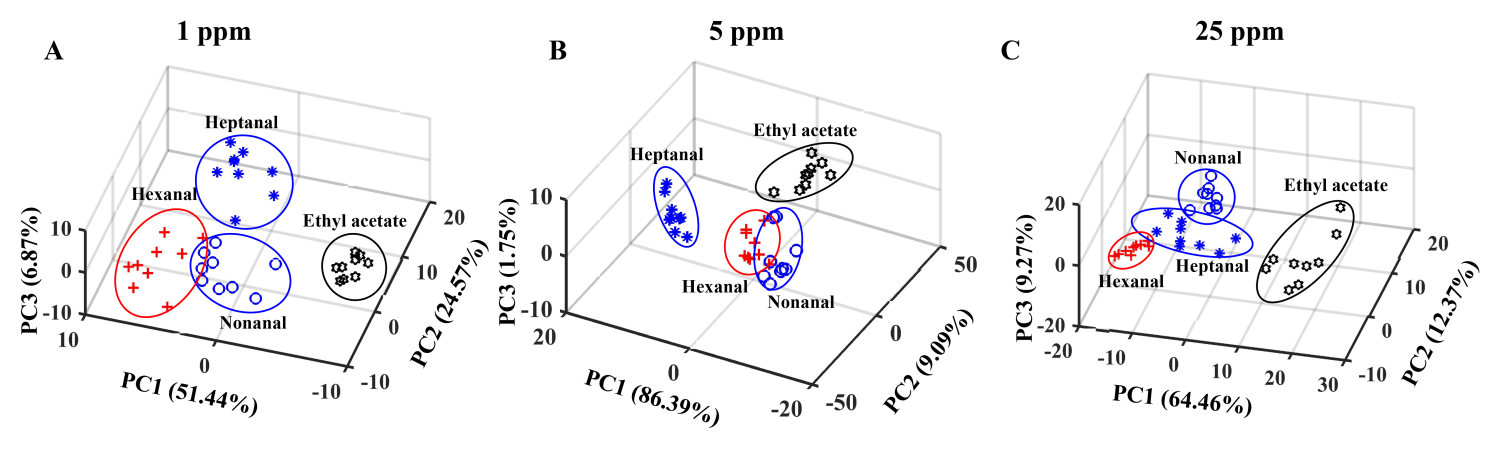


**Figure S21.** PCA plots of four different concentrations of matcha VOCs and control based on response of nine parallel trials, using the first three principal components.


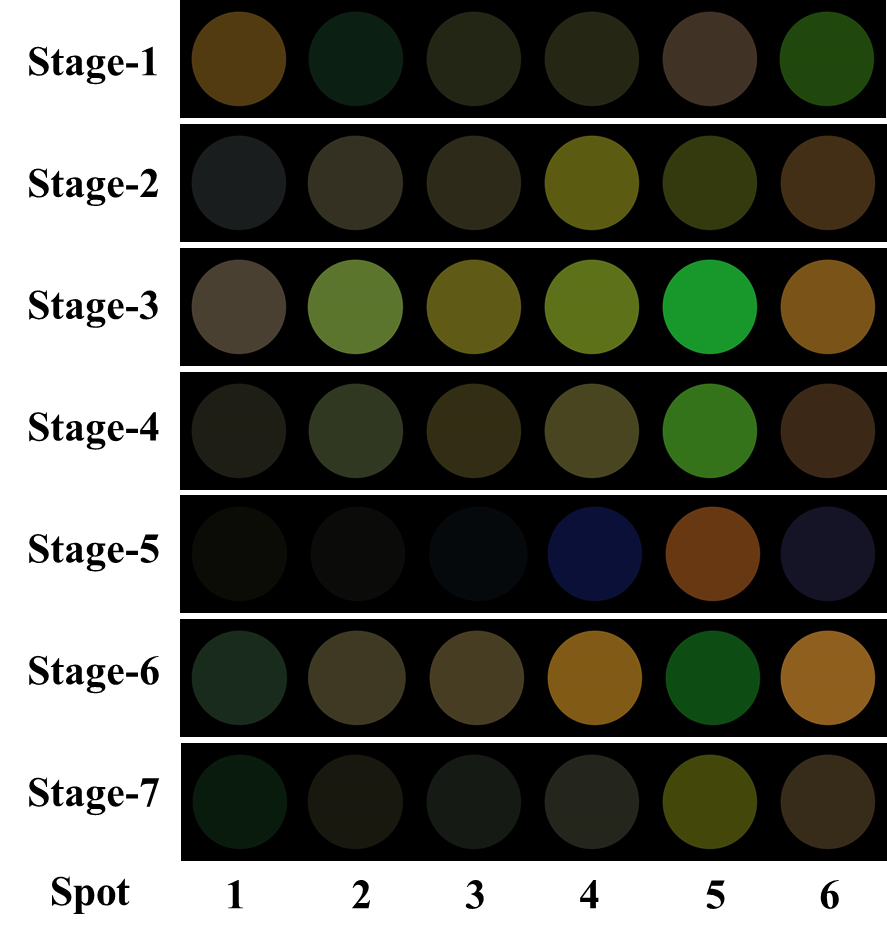


**Figure S22.** Representative colorful difference images of the Dye@ZIF-8@COF sensors for sensing VOCs released from the matcha drying samples (Spot 1-6 represent the Dye@ZIF-8@COF sensors incorporating 6 different dyes listed in Table S1).


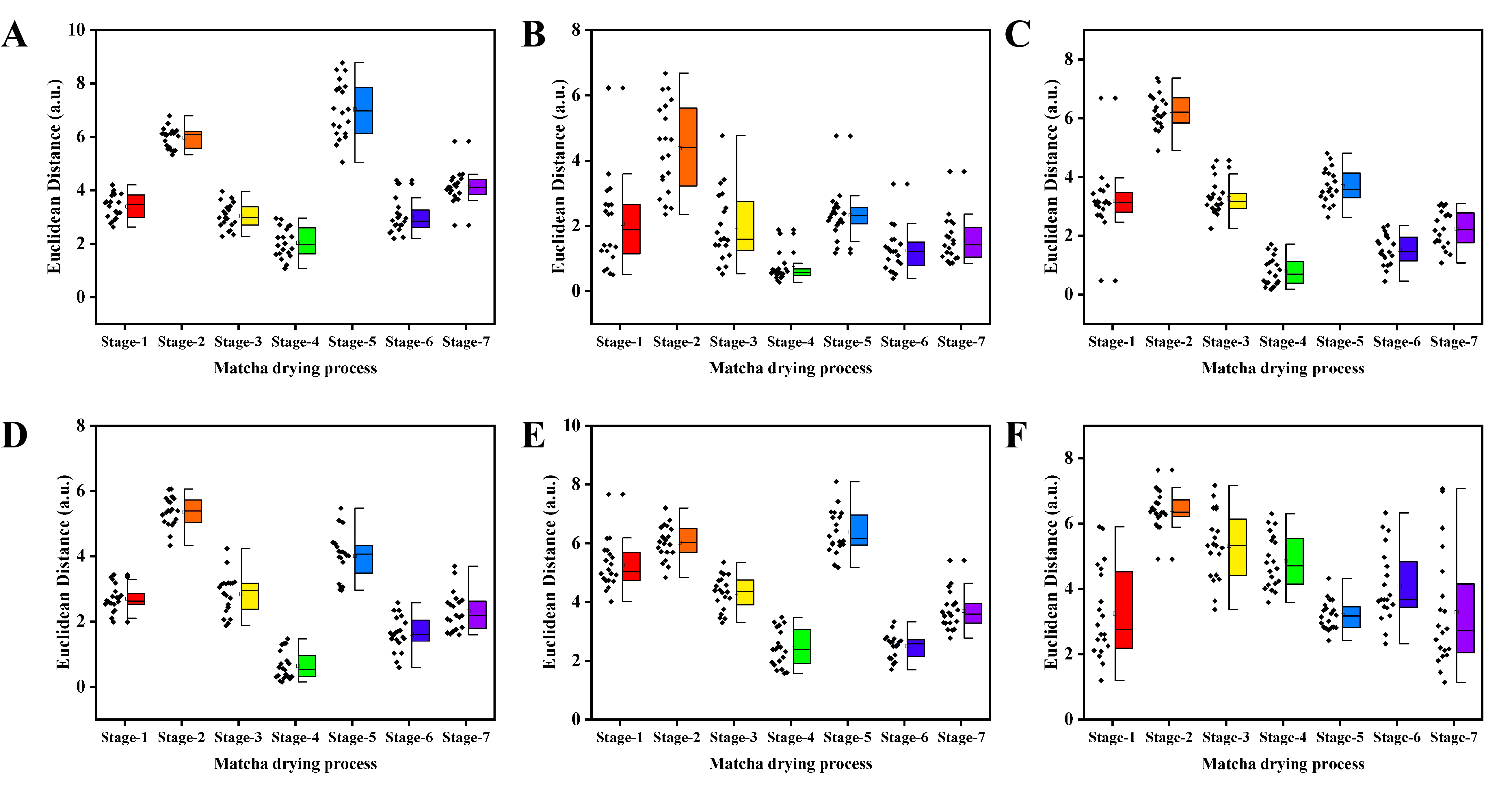


**Figure S23.** Average ED values of the A) Dye1@ZIF-8@COF, B) Dye2@ZIF-8@COF, C) Dye3@ZIF-8@COF, D) Dye4@ZIF-8@COF, E) Dye5@ZIF-8@COF, F) Dye6@ZIF-8@COF sensors for sensing VOCs released from the matcha drying samples. The six different dyes are listed in Table S1.


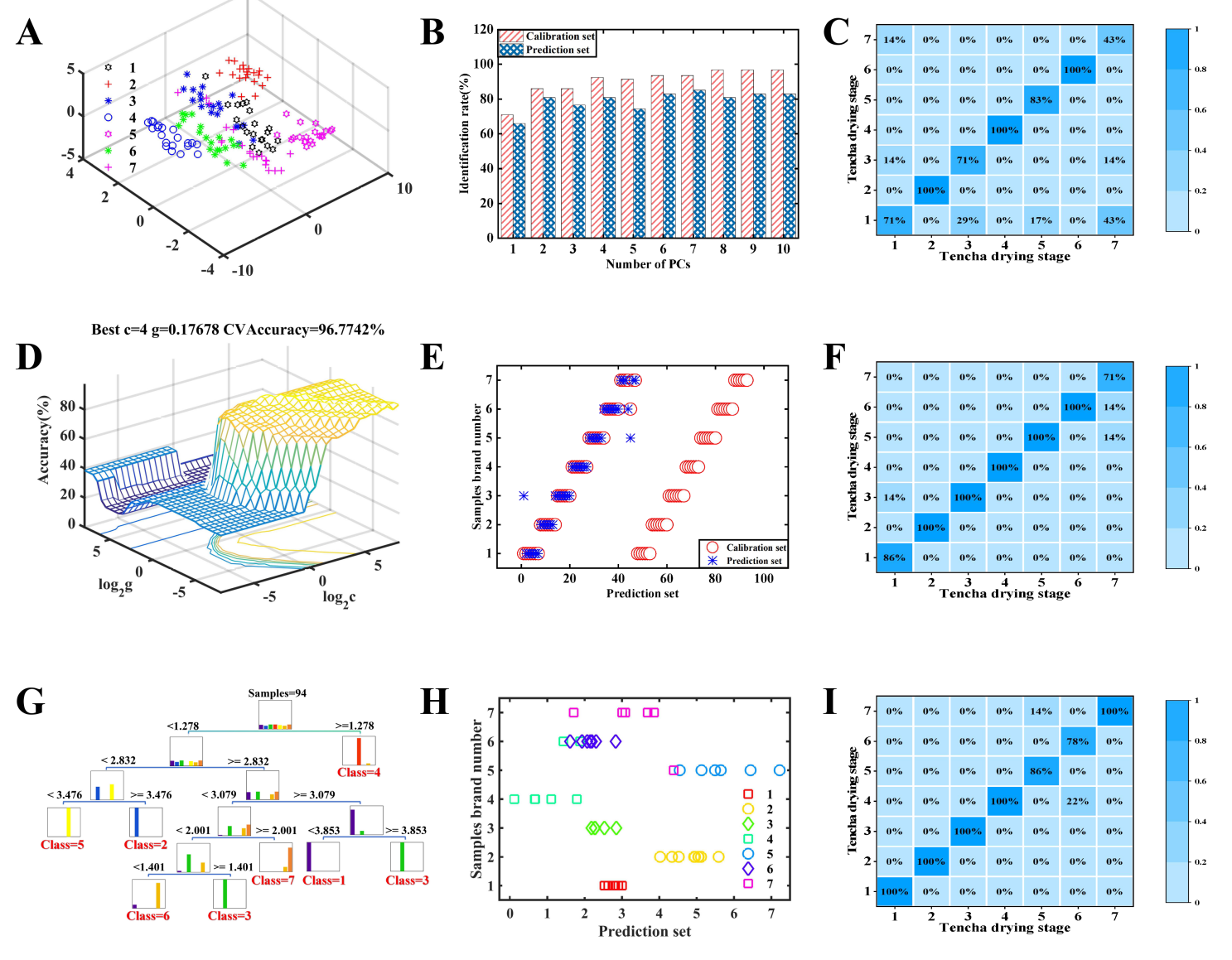


**Figure S24.** A) PCA Score diagram of the matcha drying process. B) Identification rate of LDA model with different PCs in the calibration set and prediction set. C) Confusion matrix of the optimal discriminant models for the matcha drying process by LDA. D) Contour plot of the optimal parameters of *c* and *g* by cross-validation based on SVM. E) Samples identification of the optimal parameters of PCs by cross-validation based on SVM. F) Confusion matrix of the optimal discriminant models for the matcha drying process by SVM. G) Structure diagram of CART model. H) The identification of matcha drying process in the prediction set using the CART model. I) Confusion matrix of the optimal discriminant models for the matcha drying process by CART.


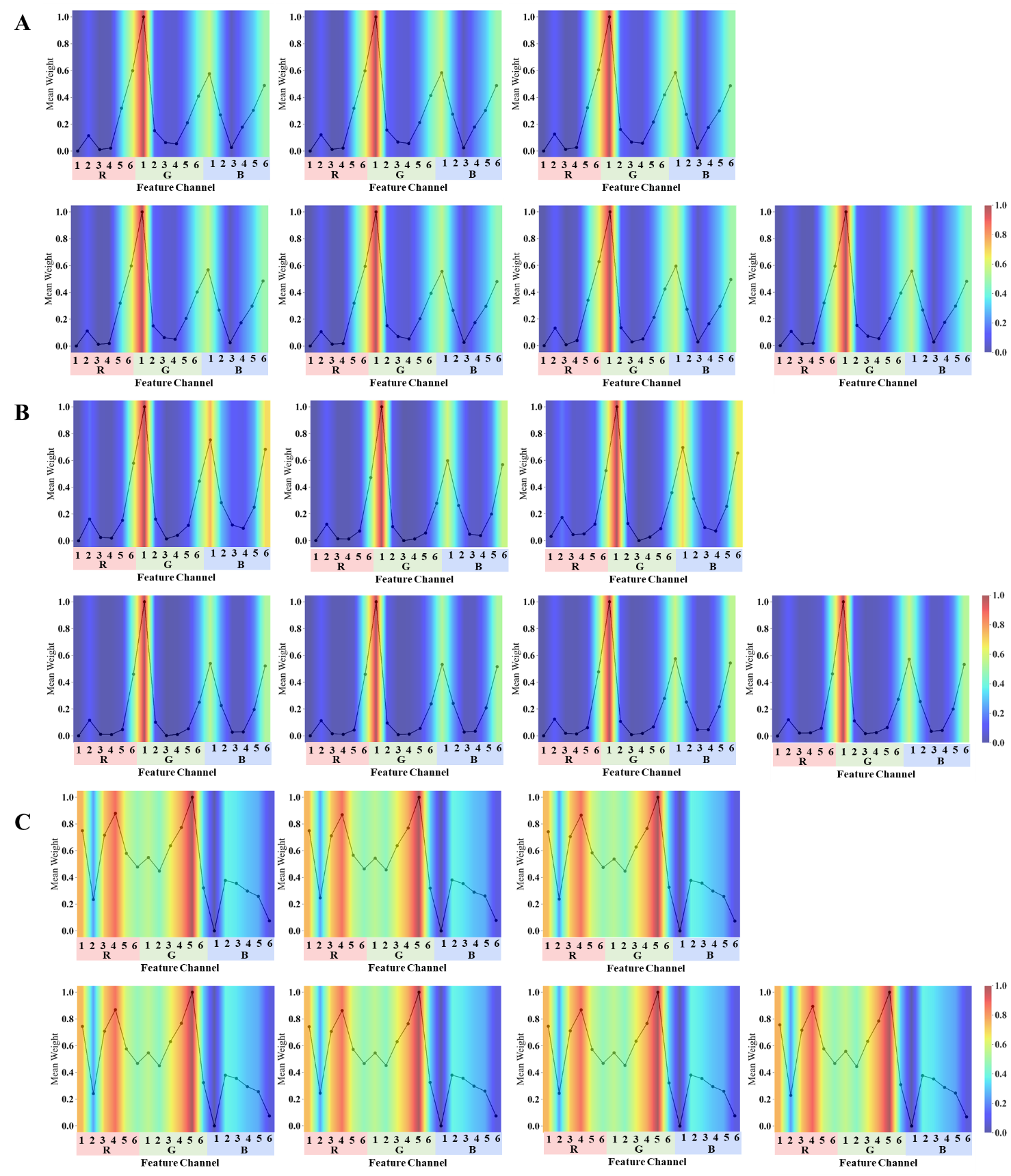


**Figure S25.** The important features of the sensor based on the Class Activation Mapping. A) Dye, B) Dye@ZIF-8, C) Dye@ZIF-8@COF. Numbers 1-6 represent the 6 different dyes listed in Table S1.

**Table S1.** Corresponding dyes used for the fabrication of different colorimetric Dye@ZIF-8@COF sensors.

| Dye | Dye-based array |
| --- | --- |
| Dye1 | 5, 10, 15, 20-Tetraphenyl-21H, 23H-porphin |
| Dye2 | 5, 10, 15, 20-Tetraphenyl-21H, 23H-porphine manganese (III) chloride |
| Dye3 | 5, 10, 15, 20-Tetrakis (4-methoxyphenyl) -21H, 23H-porphine iron (III) chloride |
| Dye4 | 5, 10, 15, 20-Tetraphenyl-21H, 23H-porphine iron (III) chloride |
| Dye5 | 5, 10, 15, 20-Tetraphenyl-21H, 23H-porphine zinc |
| Dye6 | Basic red |

**Table S2.** The limit of detection (LOD) results of sensors.

|  | LOD (ppm) | | | |
| --- | --- | --- | --- | --- |
|  | Ethyl acetate | Hexanal | Heptanal | Nonanal |
| Dye1 | 0.334 | 0.172 | 0.224 | 0.767 |
| Dye1@ZIF-8 | 0.077 | 0.044 | 0.088 | 0.370 |
| Dye1@ZIF-8@COF | **0.021** | **0.061** | **0.092** | **0.215** |
| Dye2 | 1.005 | 0.501 | 0.424 | 0.640 |
| Dye2@ZIF-8 | 1.227 | 0.577 | 0.540 | 0.617 |
| Dye2@ZIF-8@COF | **0.713** | **0.297** | **0.260** | **0.264** |
| Dye3 | 0.670 | 1.542 | 1.742 | 3.134 |
| Dye3@ZIF-8 | 0.766 | 3.336 | 2.446 | 1.162 |
| Dye3@ZIF-8@COF | **0.178** | **0.864** | **0.769** | **0.371** |
| Dye4 | 0.844 | 0.508 | 1.793 | 0.670 |
| Dye4@ZIF-8 | 0.247 | 0.178 | 0.312 | 0.236 |
| Dye4@ZIF-8@COF | **0.524** | **0.058** | **0.605** | **0.070** |
| Dye5 | 0.400 | 0.310 | 0.416 | 0.389 |
| Dye5@ZIF-8 | 0.912 | 1.102 | 1.386 | 1.080 |
| Dye5@ZIF-8@COF | **0.066** | **0.060** | **0.125** | **0.088** |
| Dye6 | 0.843 | 0.160 | 0.268 | 0.251 |
| Dye6@ZIF-8 | 0.975 | 0.080 | 0.062 | 0.115 |
| Dye6@ZIF-8@COF | **0.789** | **0.081** | **0.036** | **0.131** |

**Table S3** Comparison of the analytical capacity of the reported methods for VOCs determination.

| Substance | LOD (ppm) | Reference |
| --- | --- | --- |
| Ethyl acetate | <1 | This study |
| Hexanal |  |  |
| Heptanal |  |  |
| Nonanal |  |  |
| H_2_S | 4 | [6] |
| Acetaldehyde | 3.1 | [7] |
| Propionaldehyde | 3.5 |  |
| Formic acid | 4.6 | [8] |
| CHCl_3_ | 3.2 | [9] |

**Table S4.** DFT calculation results.

| Material | LUMO (eV) | HOMO (eV) | ΔE (eV) |
| --- | --- | --- | --- |
| NH_2_-ZIF-8 | 2.1385 | -6.8684 | 9.0069 |
| DVA | -6.9811 | 2.1385 | -9.1196 |
| TPB | -6.8641 | -6.9811 | 0.1170 |
| NH_2_-ZIF-8@DVA | -2.6275 | -6.8641 | 4.2366 |
| NH_2_-ZIF-8@DVA-TPB | -2.5897 | -2.6275 | 0.0378 |

**Table S5.** Identification and semi-quantification of VOCs in matcha drying process using HS-SPME-GC-MS.

| No. | RI ^a^ | VOCs | Matcha drying stage | | | | | | |
| --- | --- | --- | --- | --- | --- | --- | --- | --- | --- |
|  |  |  | 1 | 2 | 3 | 4 | 5 | 6 | 7 |
| V1 | 1121.15 | Cis-3-Hexenyl isovalerate | - | - | 14.37±12.49a | - | - | - | - |
| V2 | 1091.75 | (+)-2-Bornanone | 11.43±0.11a | - | - | 10.57±0.47b | - | - | - |
| V3 | 410.14 | Ethyl Acetate | 3.83±0.20d | 8.20±0.11c | 10.89±1.74b | 11.66±1.04b | 15.18±0.58a | 11.13±0.60b | 0.45±0.04e |
| V4 | 1171.66 | (E)-4,8-Dimethylnona-1,3,7-triene | - | 6.38±0.06b | - | 2.70±0.10c | - | 11.31±0.06a | 2.62±0.26c |
| V5 | 691.11 | (Z)-3-Hexen-1-ol | 13.01±0.18a | 2.10±0.00d | 1.91±0.24d | 2.96±0.07b | - | 2.55±0.08c | - |
| V6 | 763.74 | Toluene | 5.57±0.49e | 12.45±0.22ab | 13.94±2.19a | 7.56±0.25d | 11.57±0.20b | 5.14±0.21e | 9.44±0.16c |
| V7 | 645.07 | Hexanal | 2.22±0.12d | 6.26±0.02c | 8.29±1.18b | 6.44±0.43c | 8.98±0.06b | 5.86±0.40c | 11.43±0.21a |
| V8 | 608.70 | (E)-2-Hexen-1-ol | 7.14±0.16a | - | - | - | - | - | - |
| V9 | 1097.48 | (Z)-3,7-dimethyl-1,3,6-Octatriene | 0.75±0.02e | 1.78±0.02b | 0.30±0.05f | 1.26±0.13cd | 1.37±0.02c | 6.31±0.09a | 1.16±0.06d |
| V10 | 577.43 | Pentanal | 0.85±0.01d | 4.07±0.05c | 3.62±0.53c | 4.20±0.46c | 5.46±0.15b | 5.92±0.30b | 7.65±0.89a |
| V11 | 855.06 | Octanal | - | 3.30±0.02b | 4.22±0.47a | 2.78±0.06c | 3.61±0.16b | - | - |
| V12 | 645.97 | (E)-3-Hexen-1-ol | 0.16±0.01c | - | - | - | 3.02±0.05b | - | 3.41±0.27a |
| V13 | 263.15 | dimethyl-Silanediol | 1.27±0.09bc | 1.60±0.43b | 1.18±0.17c | 3.92±0.27a | 1.02±0.06c | - | - |
| V14 | 1218.85 | (E)-6-Dodecene | - | 3.01±0.07a | - | 0.33±0.03b | - | - | - |
| V15 | 2000.04 | Phytol | - | - | - | - | 2.73±0.08a | - | - |
| V16 | 455.37 | 1-methoxy-2-Propanol | 4.88±0.25a | 4.55±0.01ab | 4.65±0.73b | 4.27±0.17ab | 3.92±0.19b | 3.99±0.23b | - |
| V17 | 824.06 | (E)-3-Hexen-1-ol, acetate | - | - | - | - | - | 3.54±0.09a | - |
| V18 | 971.85 | 3,3,5-trimethyl-1-Hexene | - | - | 2.77±0.39a | - | - | - | - |
| V19 | 1066.40 | Linalool | 3.41±0.08a | 2.14±0.02b | - | 0.92±0.57c | - | - | 1.29±0.83c |
| V20 | 1237.47 | Hexanoic acid, 4-hexen-1-yl ester | 1.51±0.02c | - | 1.78±0.23b | - | - | 0.89±0.07d | 2.88±0.17a |
| V21 | 1041.37 | Eucalyptol | 0.77±0.02b | - | - | 0.93±0.04b | - | - | 3.64±0.77a |
| V22 | 1003.58 | (E)-Butanoic acid, 3-hexenyl ester | 3.44±0.13a | - | - | - | - | - | 1.29±0.08b |
| V23 | 563.77 | 1-Penten-3-ol | - | - | 1.07±0.34b | - | 2.07±0.25a | - | - |
| V24 | 1237.51 | (Z)-Hexanoic acid, 3-hexenyl ester | - | 1.51±0.05a | - | 1.43±0.07b | 0.92±0.05c | - | - |
| V25 | 754.85 | Heptanal | 1.77±0.02e | 3.42±0.07c | 3.87±0.51bc | 3.50±0.24c | 5.03±0.13a | 2.77±0.16d | 4.10±0.20b |
| V26 | 1510.49 | 2-ethyl-Butanoic acid, 1,2,3-propanetriyl ester | - | - | - | 1.55±0.42a | - | - | - |
| V27 | 550.74 | 2-methyl-Butanal | 0.34±0.05e | 2.27±0.03b | 1.76±0.27c | 2.56±0.15b | 2.34±0.09b | 1.39±0.06d | 3.05±0.34a |
| V28 | 1087.25 | D-Limonene | - | 0.93±0.01c | - | 1.24±0.07b | - | - | 1.65±0.05a |
| V29 | 1404.98 | 1-Tetradecene | 1.33±0.10a | - | - | - | 1.13±0.03b | - | - |
| V30 | 1557.31 | alpha-Farnesene | 0.78±0.05c | 1.32±0.10b | 0.29±0.05d | - | 0.64±0.02c | 2.12±0.21a | 0.34±0.02d |
| V31 | 1008.02 | Decanal | 1.99±0.10a | 1.03±0.03b | - | - | - | - | - |
| V32 | 968.34 | Nonanal | 5.19±0.15c | 6.06±0.07b | 6.12±0.75b | 5.57±0.29bc | 6.93±0.28b | 5.03±0.08c | 7.01±0.49a |
| V33 | 847.74 | 1,3,5,7-Cyclooctatetraene | 0.58±0.01cd | 0.79±0.21c | 0.25±0.04de | 1.65±0.04a | 1.25±0.44b | - | 1.55±0.06ab |
| V34 | 1228.75 | 1-Dodecanol | 0.17±0.00d | 0.10±0.00d | 0.81±0.11c | 1.16±0.06a | - | 0.95±0.05b | - |
| V35 | 1363.13 | Ethyl 2-(5-methyl-5-vinyltetrahydrofuran-2-yl)propan-2-yl carbonate | 1.74±0.05a | 1.38±0.02a | 0.80±0.12c | 1.32±0.64ab | - | - | 0.83±0.16bc |
| V36 | 1271.87 | (Z)-9-methyl-5-Undecene | 2.14±0.02a | - | - | - | - | - | - |
| V37 | 826.08 | Indole | 1.14±0.07b | 1.04±0.07b | 0.32±0.05c | 0.34±0.04c | - | 1.89±0.55a | - |
| V38 | 1090.68 | 4-ethyl-1-Octyn-3-ol | - | 1.20±0.01a | - | - | - | - | - |
| V39 | 565.37 | 2-Propenoic acid, 2-hydroxyethyl ester | - | - | - | - | - | 1.58±0.25a | - |
| V40 | 942.19 | 2-pentyl-Furan | - | - | - | - | - | - | 1.79±0.33a |
| V41 | 833.30 | 2,5-Octanedione | - | - | - | - | 0.79±0.05b | 1.21±0.19a | - |
| V42 | 1064.49 | 1-Decene | - | - | 0.95±0.12b | 0.91±0.04bc | 1.09±0.06c | 0.82±0.05a | - |
| V43 | 1079.86 | (E,E)-2,6-Dimethyl-1,3,5,7-octatetraene | - | - | - | - | - | 1.45±0.13a | - |
| V44 | 1565.99 | (E)-3,7,11-trimethyl-1,6,10-Dodecatrien-3-ol | 0.27±0.02b | 1.03±0.12a | 0.14±0.05b | - | - | 1.18±0.40a | - |
| V45 | 1474.49 | Sulfurous acid, butyl 2-ethylhexyl ester | 0.05±0.01d | - | - | 0.33±0.02c | 0.72±0.04b | 1.28±0.02a | - |
| V46 | 669.34 | 2,5-dimethyl-Pyrazine | - | 0.18±0.03c | 0.19±0.03c | 0.18±0.07c | 0.10±0.01cd | 0.40±0.05b | 1.76±0.20a |
| V47 | 660.48 | 1-ethyl-1H-Pyrrole | - | - | - | - | - | - | 1.64±0.23a |
| V48 | 1099.01 | .alpha.-Pinene | - | 0.93±0.01a | - | - | - | - | - |
| V49 | 1762.71 | 1-chloro-7-Heptadecene | - | - | - | - | 0.90±0.06a | - | - |
| V50 | 820.81 | o-Xylene | 0.46±0.01a | 0.91±0.03d | 1.14±0.18c | 1.25±0.05bc | 1.32±0.05b | 0.88±0.03d | 2.29±0.11a |
| V51 | 1064.49 | 1-Decene | - | 0.89±0.02a | - | - | - | - | - |
| V52 | 834.36 | 2,6-dimethyl-Cyclohexanone | - | - | - | - | - | - | 1.48±0.33a |
| V53 | 853.13 | 1-Octanol | 0.53±0.02b | - | - | 0.92±0.26a | - | - | - |

^a^ RI: retention index

**Table S6.** Eigenvalue and cumulate contribution rates of the principal components from VOCs detection in matcha drying process using HS-SPME-GC-MS.

| Principal Component | Eigenvalue | Percentage of Variance (%) | Cumulative (%) |
| --- | --- | --- | --- |
| 1^st^ | 14.245 | 26.878 | 26.878 |
| 2^nd^ | 11.622 | 21.928 | 48.806 |
| 3^rd^ | 9.415 | 17.764 | 66.57 |
| 4^th^ | 7.109 | 13.414 | 79.984 |
| 5^th^ | 5.254 | 9.913 | 89.897 |
| 6^th^ | 4.04 | 7.623 | 97.519 |

**Table S7.** VIP value and data distribution of the characters indicators during matcha drying process using HS-SPME-GC-MS.

| NO. | VIP value | Mean | SD ^a^ | Minimum | Maximum | Significance | CV ^b^ |
| --- | --- | --- | --- | --- | --- | --- | --- |
| V1 | 3.89157 | 2.0522 | 1.41626 | 0 | 23.01 | 0 | 69.01% |
| V2 | 3.45241 | 3.1423 | 1.11264 | 0 | 11.54 | 0 | 35.41% |
| V3 | 3.23975 | 8.7623 | 1.05815 | 0.4 | 15.64 | 0.002 | 12.08% |
| V4 | 3.06135 | 3.2883 | 0.87514 | 0 | 11.38 | 0.005 | 26.61% |
| V5 | 2.81434 | 3.2172 | 0.92634 | 0 | 13.21 | 0 | 28.79% |
| V6 | 2.77125 | 9.3815 | 0.72884 | 5 | 16.42 | 0.001 | 7.77% |
| V7 | 2.27032 | 7.0663 | 0.60591 | 2.09 | 11.68 | 0 | 8.57% |
| V8 | 2.26574 | 1.0196 | 0.55857 | 0 | 7.27 | 0 | 54.78% |
| V9 | 2.09045 | 1.8491 | 0.4192 | 0.27 | 6.39 | 0.012 | 22.67% |
| V10 | 1.9917 | 4.5367 | 0.44793 | 0.85 | 8.28 | 0.002 | 9.87% |
| V11 | 1.98924 | 1.9873 | 0.39632 | 0 | 4.76 | 0 | 19.94% |
| V12 | 1.97018 | 0.9411 | 0.32338 | 0 | 3.61 | 0 | 34.36% |
| V13 | 1.9411 | 1.2843 | 0.27544 | 0 | 4.08 | 0.001 | 21.45% |
| V14 | 1.88104 | 0.4779 | 0.2329 | 0 | 3.09 | 0.014 | 48.73% |
| V15 | 1.8028 | 0.3893 | 0.2133 | 0 | 2.79 | 0 | 54.79% |
| V16 | 1.78785 | 3.7532 | 0.3549 | 0 | 5.48 | 0.001 | 9.46% |
| V17 | 1.74398 | 0.5063 | 0.27737 | 0 | 3.61 | 0 | 54.78% |
| V18 | 1.72823 | 0.396 | 0.21855 | 0 | 3.22 | 0 | 55.19% |
| V19 | 1.67234 | 1.1085 | 0.27753 | 0 | 3.5 | 0.002 | 25.04% |
| V20 | 1.6477 | 1.0076 | 0.23087 | 0 | 3.07 | 0.001 | 22.91% |
| V21 | 1.61709 | 0.7622 | 0.28053 | 0 | 4.43 | 0.009 | 36.81% |
| V22 | 1.59808 | 0.6753 | 0.27121 | 0 | 3.54 | 0 | 40.16% |
| V23 | 1.45631 | 0.4477 | 0.17164 | 0 | 2.34 | 0.001 | 38.34% |
| V24 | 1.43941 | 0.55 | 0.14726 | 0 | 1.57 | 0 | 26.77% |
| V25 | 1.42488 | 3.4933 | 0.21761 | 1.75 | 5.16 | 0.005 | 6.23% |
| V26 | 1.37025 | 0.2212 | 0.1246 | 0 | 2.03 | 0 | 56.33% |
| V27 | 1.36607 | 1.9597 | 0.18759 | 0.29 | 3.29 | 0.004 | 9.57% |
| V28 | 1.36386 | 0.5455 | 0.14743 | 0 | 1.7 | 0.001 | 27.03% |
| V29 | 1.33018 | 0.3509 | 0.12485 | 0 | 1.42 | 0.001 | 35.58% |
| V30 | 1.31182 | 0.783 | 0.1506 | 0 | 2.31 | 0.008 | 19.23% |
| V31 | 1.30809 | 0.432 | 0.16319 | 0 | 2.06 | 0 | 37.78% |
| V32 | 1.29404 | 5.9875 | 0.17671 | 4.96 | 7.56 | 0.005 | 2.95% |
| V33 | 1.28706 | 0.8684 | 0.13666 | 0 | 1.69 | 0 | 15.74% |
| V34 | 1.27468 | 0.4547 | 0.10342 | 0 | 1.22 | 0.003 | 22.74% |
| V35 | 1.26319 | 0.8668 | 0.14714 | 0 | 2.06 | 0 | 16.98% |
| V36 | 1.24231 | 0.3064 | 0.1678 | 0 | 2.17 | 0 | 54.77% |
| V37 | 1.21203 | 0.6755 | 0.15059 | 0 | 2.23 | 0 | 22.29% |
| V38 | 1.197 | 0.1712 | 0.09377 | 0 | 1.21 | 0.004 | 54.77% |
| V39 | 1.16308 | 0.2252 | 0.12456 | 0 | 1.79 | 0.001 | 55.31% |
| V40 | 1.12827 | 0.2558 | 0.14194 | 0 | 1.98 | 0 | 55.49% |
| V41 | 1.12813 | 0.2863 | 0.10512 | 0 | 1.41 | 0.004 | 36.72% |
| V42 | 1.11857 | 0.5393 | 0.10616 | 0 | 1.14 | 0.001 | 19.68% |
| V43 | 1.11789 | 0.2074 | 0.11395 | 0 | 1.53 | 0 | 54.94% |
| V44 | 1.09594 | 0.3757 | 0.10975 | 0 | 1.61 | 0.004 | 29.21% |
| V45 | 1.0954 | 0.3404 | 0.10195 | 0 | 1.3 | 0.007 | 29.95% |
| V46 | 1.08119 | 0.401 | 0.12775 | 0 | 1.99 | 0 | 31.86% |
| V47 | 1.08106 | 0.234 | 0.12917 | 0 | 1.89 | 0.001 | 55.20% |
| V48 | 1.05752 | 0.1335 | 0.07314 | 0 | 0.95 | 0.007 | 54.79% |
| V49 | 1.03796 | 0.1289 | 0.07073 | 0 | 0.96 | 0.002 | 54.87% |
| V50 | 1.03322 | 1.1783 | 0.11882 | 0.45 | 2.36 | 0.002 | 10.08% |
| V51 | 1.03179 | 0.1272 | 0.06968 | 0 | 0.91 | 0 | 54.78% |
| V52 | 1.02221 | 0.2109 | 0.11776 | 0 | 1.72 | 0 | 55.84% |
| V53 | 1.00146 | 0.2069 | 0.07879 | 0 | 1.11 | 0 | 38.08% |

^a^ SD: Standard deviation

^b^ CV: Coefficient of variation

**Table S8.** Loading values of Principal Component Analysis from VOCs detection in matcha drying process using HS-SPME-GC-MS.

| NO. | 1 | 2 | 3 | 4 | 5 | 6 |
| --- | --- | --- | --- | --- | --- | --- |
| V1 | 0.033 | 0.062 | -0.232 | -0.167 | -0.029 | -0.768 |
| V2 | -0.498 | -0.606 | -0.135 | -0.25 | 0.486 | 0.256 |
| V3 | -0.141 | 0.713 | -0.642 | -0.153 | 0.099 | 0.114 |
| V4 | -0.268 | 0.595 | 0.535 | 0.496 | 0.154 | 0.114 |
| V5 | -0.756 | -0.578 | 0.119 | -0.256 | -0.062 | 0.035 |
| V6 | 0.49 | 0.076 | -0.592 | 0.27 | -0.367 | -0.399 |
| V7 | 0.964 | 0.22 | 0.015 | 0.022 | -0.028 | -0.101 |
| V8 | -0.605 | -0.648 | 0.15 | -0.354 | -0.226 | 0.098 |
| V9 | -0.314 | 0.724 | 0.556 | 0.107 | 0.102 | 0.194 |
| V10 | 0.762 | 0.464 | 0.363 | 0.127 | 0.089 | 0.157 |
| V11 | 0.177 | 0.225 | -0.907 | 0.17 | -0.06 | -0.237 |
| V12 | 0.805 | -0.062 | 0.144 | -0.279 | -0.321 | 0.373 |
| V13 | -0.187 | -0.253 | -0.657 | 0.157 | 0.634 | 0.167 |
| V14 | -0.141 | -0.048 | -0.233 | 0.928 | -0.231 | 0.081 |
| V15 | 0.289 | 0.322 | -0.456 | -0.407 | -0.429 | 0.498 |
| V16 | -0.779 | 0.163 | -0.567 | -0.007 | -0.03 | -0.094 |
| V17 | -0.356 | 0.71 | 0.586 | -0.044 | 0.121 | 0.024 |
| V18 | 0.083 | 0.094 | -0.32 | -0.187 | -0.04 | -0.902 |
| V19 | -0.402 | -0.804 | 0.145 | 0.238 | -0.184 | 0.196 |
| V20 | 0.362 | -0.381 | 0.621 | -0.269 | -0.101 | -0.488 |
| V21 | 0.626 | -0.51 | 0.53 | -0.026 | 0.182 | 0.086 |
| V22 | -0.321 | -0.767 | 0.363 | -0.337 | -0.224 | 0.099 |
| V23 | 0.309 | 0.349 | -0.586 | -0.479 | -0.424 | 0.022 |
| V24 | 0.019 | 0.043 | -0.636 | 0.568 | 0.211 | 0.467 |
| V25 | 0.814 | 0.343 | -0.42 | -0.033 | -0.124 | 0.093 |
| V26 | -0.008 | -0.101 | -0.334 | 0.044 | 0.871 | 0.23 |
| V27 | 0.857 | 0.137 | -0.144 | 0.351 | 0.243 | 0.156 |
| V28 | 0.576 | -0.381 | 0.182 | 0.514 | 0.432 | 0.187 |
| V29 | -0.298 | -0.311 | -0.199 | -0.583 | -0.492 | 0.435 |
| V30 | -0.494 | 0.563 | 0.479 | 0.279 | -0.332 | 0.132 |
| V31 | -0.645 | -0.636 | 0.047 | 0.115 | -0.378 | 0.121 |
| V32 | 0.841 | -0.066 | -0.204 | 0.009 | -0.335 | 0.091 |
| V33 | 0.607 | -0.39 | -0.227 | 0.057 | 0.323 | 0.519 |
| V34 | -0.307 | 0.384 | -0.071 | -0.082 | 0.797 | -0.312 |
| V35 | -0.314 | -0.821 | -0.13 | 0.315 | 0.169 | -0.084 |
| V36 | -0.605 | -0.648 | 0.15 | -0.354 | -0.226 | 0.098 |
| V37 | -0.78 | 0.299 | 0.458 | 0.223 | -0.043 | 0.003 |
| V38 | -0.137 | -0.036 | -0.193 | 0.912 | -0.327 | 0.055 |
| V39 | -0.358 | 0.708 | 0.588 | -0.04 | 0.117 | 0.025 |
| V40 | 0.741 | -0.329 | 0.572 | 0.037 | 0.001 | 0.002 |
| V41 | -0.155 | 0.835 | 0.266 | -0.276 | -0.147 | 0.316 |
| V42 | 0.064 | 0.679 | -0.468 | -0.455 | 0.309 | -0.062 |
| V43 | -0.358 | 0.711 | 0.589 | -0.042 | 0.119 | 0.025 |
| V44 | -0.519 | 0.463 | 0.359 | 0.555 | -0.193 | -0.009 |
| V45 | -0.219 | 0.82 | 0.242 | -0.272 | 0.097 | 0.364 |
| V46 | 0.731 | -0.165 | 0.646 | 0.092 | 0.063 | -0.034 |
| V47 | 0.743 | -0.325 | 0.571 | 0.039 | 0.005 | 0.002 |
| V48 | -0.137 | -0.036 | -0.192 | 0.912 | -0.327 | 0.054 |
| V49 | 0.289 | 0.322 | -0.456 | -0.407 | -0.428 | 0.497 |
| V50 | 0.958 | -0.046 | 0.21 | 0.012 | 0.147 | 0.031 |
| V51 | -0.137 | -0.036 | -0.193 | 0.911 | -0.326 | 0.054 |
| V52 | 0.736 | -0.329 | 0.569 | 0.036 | 0 | 0.002 |
| V53 | -0.339 | -0.452 | -0.233 | -0.144 | 0.701 | 0.271 |

**Table S9.** Representative indicators of the six principal components using HS-SPME-GC-MS data.

| PCs ^a^ | Representative Indicators |
| --- | --- |
| 1 | V2, V5, V7, V8, V10, V12, V16, V21, V25, V27, V28, V31, V32, V33, V36, V37, V40, V44, V46, V47, V50 |
| 2 | V2, V3, V5, V8, V9, V17, V19, V22, V30, V31, V35, V36, V39, V41, V42, V43, V45 |
| 3 | V3, V4, V6, V9, V11, V13, V16, V17, V20, V21, V23, V24, V39, V40, V43, V46, V47, V52 |
| 4 | V14, V24, V28, V29, V38, V44, V48, V51 |
| 5 | V13, V26, V34, V53 |
| 6 | V1, V18, V33 |

^a^ PCs: Principal components.

**Table S10.** Characterization of main VOCs identified from the matcha drying process using HS-SPME-GC-MS.

| NO. | RI ^a^ | VOCs | Matcha drying stage | | | | | | | Structure |
| --- | --- | --- | --- | --- | --- | --- | --- | --- | --- | --- |
|  |  |  | 1 | 2 | 3 | 4 | 5 | 6 | 7 |  |
| 1 | 410.14 | Ethyl Acetate | 3.83±0.20a | 8.20±0.11c | 10.89±1.74b | 11.66±1.04b | 15.18±0.58a | 11.13±0.60b | 0.45±0.04e | 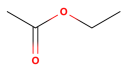 |
| 2 | 645.07 | Hexanal | 2.22±0.12d | 6.26±0.02c | 8.29±1.18b | 6.44±0.43c | 8.98±0.06b | 5.86±0.40c | 11.43±0.21a | 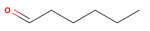 |
| 3 | 754.85 | Heptanal | 1.77±0.02e | 3.42±0.07c | 3.87±0.51bc | 3.50±0.24c | 5.03±0.13a | 2.77±0.16d | 4.10±0.20b | 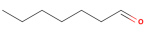 |
| 4 | 968.34 | Nonanal | 5.19±0.15c | 6.06±0.07b | 6.12±0.75b | 5.57±0.29bc | 6.93±0.28b | 5.03±0.08c | 7.01±0.49a | 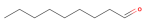 |

^a^ RI: retention index

**Table S11.** Eigenvalue and cumulate contribution rates of the principal components from sensors data.

| Principal Component | Eigenvalue | Percentage of Variance (%) | Cumulative (%) |
| --- | --- | --- | --- |
| 1^st^ | 10.2230 | 56.7942 | 56.7942 |
| 2^nd^ | 2.0072 | 11.1510 | 67.945 |
| 3^rd^ | 1.3022 | 7.2342 | 75.1794 |
| 4^th^ | 1.1691 | 6.4951 | 81.6745 |
| 5^th^ | 0.7998 | 4.4434 | 86.1179 |
| 6^th^ | 0.5500 | 3.0557 | 89.1736 |
| 7^th^ | 0.4492 | 2.4954 | 91.6690 |
| 8^th^ | 0.3845 | 2.1360 | 93.8050 |
| 9^th^ | 0.2946 | 1.6364 | 95.441 |
| 10^th^ | 0.2534 | 1.4080 | 96.8494 |

**Table S12.** The identification results of models in the calibration and prediction set by Dye, Dye@ZIF-8, and Dye@ZIF-8@COF.

| Model | Sensors | Calibration set accuracy (%) | Prediction set accuracy (%) |
| --- | --- | --- | --- |
| LDA | Dye | 61.29 | 57.45 |
|  | Dye@ZIF-8 | 79.76 | 71.43 |
|  | **Dye@ZIF-8@COF** | **86.02** | **80.85** |
| SVM | Dye | 90.48 | 80.36 |
|  | Dye@ZIF-8 | 98.92 | 85.11 |
|  | **Dye@ZIF-8@COF** | **98.92** | **93.62** |
| CART | Dye | 85.11 | 78.26 |
|  | Dye@ZIF-8 | 89.36 | 86.96 |
|  | **Dye@ZIF-8@COF** | **94.68** | **93.48** |
| CNN | Dye | 100.00 | 89.36 |
|  | Dye@ZIF-8 | 96.77 | 85.11 |
|  | **Dye@ZIF-8@COF** | **100.00** | **95.74** |

References

[1] F. Pérez-Rodríguez, E. Gómez-García, *Comput. Electron. Agric.* **2019**, *163*, 104880.

[2] A. Winkler, M. Rauwolf, J. H. Sterba, P. Wobrauschek, C. Streli, A. Turyanskaya, *J. Sci. Food Agric.* **2020**, *100*, 4226.

[3] M. Esteki, N. Memarbashi, J. Simal-Gandara, *J. Food Compos. Anal.* **2022**, *106*, 104321.

[4] S. Sharma, K. C. Sumesh, P. Sirisomboon, *Measurement* **2022**, *189*, 110464.

[5] F. Cosme, J. Milheiro, J. Pires, F. I. Guerra-Gomes, L. Filipe-Ribeiro, F. M. Nunes, *Food Control* **2021**, *125*, 107979.

[6] S. Das, P. Sahoo, *Sens. Actuators, B* **2019**, *291*, 287.

[7] L. Yang, C. Huangfu, Y. Wang, Y. Qin, A. Qin, L. Feng, *Talanta* **2024**, *280*, 126716.

[8] X. Xia, R. Wu, L. Zhang, X. Chen, Y. Yan, J. Yin, J. Ren, H. Li, J. Yin, Z. Xue, L. Yi, T. Wang, *Anal. Chem.* **2023**, *95*, 12313.

[9] H. Im, J. Choi, H. Lee, Z. Y. Al Balushi, D.H. Park, S. Kim, *ACS Sens.* **2023**, *8*, 3370.

1. ^*^ Corresponding author. E-mail address: oyqyf@ujs.edu.cn (Q. Ouyang), yujie.ma@manchester.ac.uk (Y. Ma), zhonghua-liu@hunau.edu.cn (Z. Liu) [↑](#footnote-ref-1)
2. [↑](#footnote-ref-2)
3. [↑](#footnote-ref-3)
